# Characterization of splenic MRC1^hi^MHCII^lo^ and MRC1^lo^MHCII^hi^ cells from the monocyte/macrophage lineage of White Leghorn chickens

**DOI:** 10.1186/s13567-020-00795-9

**Published:** 2020-05-27

**Authors:** Keesun Yu, Min Jeong Gu, Young Jin Pyung, Ki-Duk Song, Tae Sub Park, Seung Hyun Han, Cheol-Heui Yun

**Affiliations:** 1 Department of Agricultural Biotechnology and Research Institute of Agriculture and Life Sciences, Seoul National University, Seoul, 08826 Republic of Korea; 2 Department of Animal Biotechnology, College of Agricultural and Life Sciences, Jeonbuk National University, Jeonju, 54896 Republic of Korea; 3 Graduate School of International Agricultural Technology and Institute of Green-Bio Science and Technology, Seoul National University, Pyeongchang-gun, Seoul, Gangwon-do 25354 Republic of Korea; 4 Department of Oral Microbiology and Immunology, DRI, and BK21 Plus Programme, School of Dentistry, Seoul National University, Seoul, 08826 Republic of Korea; 5 Center for Food Bioconvergence, Seoul National University, Seoul, 08826 Republic of Korea

## Abstract

Monocytes/macrophages, which are found in a variety of organs, maintain tissue homeostasis at a steady state and act as the first line of defence during pathogen-induced inflammation in the host. Most monocyte/macrophage lineage studies in chickens have been largely performed using cell lines, while few studies using primary cells have been conducted. In the present study, the phenotypic and functional characteristics of splenic monocyte/macrophage lineage cells during steady state and inflammatory conditions were examined. Splenic monocyte/macrophage lineage cells could be identified as MRC1^lo^MHCII^hi^ and MRC1^hi^MHCII^lo^ cells based on their surface expression of MRC1 and MHCII. In the steady state, MRC1^lo^MHCII^hi^ cells were more frequently found among MRC1^+^ cells. MRC1^lo^MHCII^hi^ cells expressed a higher number of antigen-presenting molecules (MHCII, MHCI, and CD80) than MRC1^hi^MHCII^lo^ cells. In contrast, MRC1^hi^MHCII^lo^ cells showed better phagocytic and CCR5-dependent migratory properties than MRC1^lo^MHCII^hi^ cells. Furthermore, MRC1^hi^MHCII^lo^ cells infiltrated the spleen in vivo and then became MRC1^lo^MHCII^hi^ cells. During lipopolysaccharide (LPS)-induced inflammatory conditions that were produced via intraperitoneal (i.p.) injection, the proportion and absolute number of MRC1^hi^MHCII^lo^ cells were increased in the spleen. Uniquely, inflammation induced the downregulation of MHCII expression in MRC1^hi^MHCII^lo^ cells. The major source of inflammatory cytokines (IL-1β, IL-6, and IL-12) was MRC1^lo^MHCII^hi^ cells. Furthermore, MRC1^hi^MHCII^lo^ cells showed greater bactericidal activity than MRC1^lo^MHCII^hi^ cells during LPS-induced inflammation. Collectively, these results suggest that two subsets of monocyte/macrophage lineage cells exist in the chicken spleen that have functional differences.

## Introduction

Monocytes/macrophages, which comprise the majority of mononuclear phagocytes, are derived from bone marrow precursors [1]. Macrophages are located in various organs and seeded during the prenatal stage, and they are maintained through self-proliferation or, to some extent, via the infiltration of circulating monocytes [2]. Thus, macrophages exist in several types of tissues under steady-state conditions, in which they clear apoptotic and senescent cells [3, 4]. Furthermore, macrophages are rapidly recruited locally via chemokine signals and are generated by the differentiation of circulating monocytes in response to inflammation or pathogen invasion [5].

Monocytes/macrophages are part of the innate immune system and function as the first line of defence in the host through various effector functions. They express several kinds of pattern recognition receptors (PRRs), including Toll-like receptors (TLRs) and C-type lectin receptors that recognize pathogens [6], and then phagocytose and clear the pathogen by lysosomal acidification [7]. Once activated, monocytes/macrophages release pro-inflammatory cytokines such as IL-1β, IL-6, and IL-12 [8].

Among lymphoid organs, the mammalian spleen is known to contain various types of mononuclear phagocyte subsets that are defined by phenotype, function and localization [9]. However, the spleen of chickens differs from that of mammals in both structure and function [10]. It has been reported that red pulp monocyte/macrophage lineage cells in spleen from chicken express MHCII and show a high phagocytosis ability that is similar to that of mammalian red pulp macrophages [11]. In addition, monocyte/macrophage lineage cells are also found in chicken ellipsoids [11, 12], which are analogous to the mammalian marginal zone.

Chicken mononuclear phagocytes include monocytes, and macrophage-like and dendritic cell (DC)-like cells [13]. The phenotype and function of macrophage- and DC-like cells are poorly defined because of a lack of appropriate reagents. However, in vitro culture of mononuclear phagocytes demonstrated that KUL01, which targets mannose receptor C-type 1 (MRC1), the homologue of the mammalian mannose receptor [14], can be used as a representative marker of monocyte/macrophage lineage cells, whereas 8F2 (putative chicken CD11c) can be used as a marker of DC-like cells [12]. Furthermore, comparative profiling of gene expression in splenic mononuclear phagocytes was performed between chickens and mammals, demonstrating that MRC1^+^ and CD11c^+^ cells in the spleen in chicken are distinct phagocytic populations similar to macrophages and DCs, respectively, which are the analogous mammalian counterparts [13].

Chicken monocyte/macrophage lineage cells expressing MRC1 have been found to exhibit features similar to those in mammals, including morphological features [15], plasticity [16], and capability for phagocytosis [17, 18]. Furthermore, they express several TLRs, and the activation of TLRs by their ligands induces bacterial lysis [19] and pro-inflammatory cytokine secretion [20], suggesting that they induce a similar response during inflammation compared to that of mammals. However, most studies of the functional aspects of chicken monocyte/macrophage lineage cells have been limited to in vitro experiments using cell lines (i.e., HD11 and MQ-NCSU) and bone marrow- or monocyte-derived macrophages [21–24]. Moreover, few studies have addressed questions related to defining the function of chicken primary monocyte/macrophage lineage cells.

Due to the poorly developed lymphatic system and lack of draining lymph nodes in chickens, the spleen is considered the most important secondary immune organ in chickens [25]. Therefore, characterization of splenic monocyte/macrophage lineage cells will be helpful to investigate the regulation of systemic inflammation in chickens. However, how monocyte/macrophage lineage cells are maintained in the steady state and respond to inflammatory conditions in the spleen has not been well defined. Therefore, the aim of the present study was to examine the phenotype, function, and maintenance of splenic monocyte/macrophage lineage cells during steady state and inflammatory conditions.

## Materials and methods

### Experimental animals and their treatment

Fertilized eggs of White Leghorn chickens obtained from the University Animal Farm (Pyeongchang campus, Seoul National University, Korea) were incubated in a 37.5–38 °C incubator for 21 days. The hatched chicks were housed under conventional conditions and allowed free access to food and water until they were 3 weeks old without any vaccination. All chickens used were 3 weeks of age. All experimental procedures using fertilized eggs and chickens were performed with the approval of the Institutional Animal Care and Use Committee of Seoul National University (IACUC No., SNU-150327-2).

### Single-cell dissociation

The spleen, caecal tonsil, lung, thymus, and bursa of Fabricius from 3-week-old chickens were prepared as previously mentioned [26, 27] with minor modifications. Briefly, the organs were minced and washed with RPMI-1640 (Thermo Fisher Scientific, Waltham, MA, USA) supplemented with 5% heat-inactivated fetal bovine serum (FBS; Thermo Fisher Scientific) to obtain a single-cell suspension. To isolate the peripheral blood mononuclear cells (PBMCs), blood was collected through the brachial wing vein using a 5 mL syringe equipped with a 26-gauge (0.45 mm diameter) needle and stored in BD Vacutainer tubes coated with sodium heparin (BD Biosciences, Franklin Lakes, NJ, USA). The blood was diluted in phosphate-buffered saline (PBS) at a 1:1 ratio, and PBMCs were isolated by density gradient centrifugation for 20 min at 400 × *g* without braking using Ficoll-Paque (Sigma-Aldrich, St Louis, MO, USA) and washed with PBS containing 5% FBS. To obtain bone marrow cells, femurs and tibias were obtained, both ends of the bone were cut, and the marrow was flushed out with RPMI-1640 media supplemented with 5% FBS. Clusters within the marrow cell suspension were disaggregated using a 70 μm nylon cell strainer (Corning Inc., Corning, NY, USA). Red blood cells in the cell suspension were lysed using ACK buffer (Corning Inc.) for 5–7 min at 4 °C and then washed. A single cell suspension was passed through a 70 μm cell strainer. The total cell numbers were determined using an automatic cell counter. The absolute number of cells in the target cell population was calculated by multiplying the percentage of target cells (Additional file 1A) by the total cell number for each organ examined [26, 28].

### Flow cytometric analysis

For cell surface staining, 0.5−1 × 10^6^ cells (splenocytes or sorted cells) were stained with fluorochrome-conjugated monoclonal antibodies in PBS containing 5% FBS for 20 min at 4 °C in the dark. The primary antibodies used were anti-chicken MHCII-FITC (2G11), MRC1-PE (KUL01), CD3-Pacific Blue (CT3), Bu-1-Alexa-647 (AV20), MHCI-Biotin (F21-2) (all from Southern Biotechnology, Birmingham, AL, USA), and CD80 (IAH:F864:DC7) (BioRad Laboratories, Hercules, CA, USA). After incubation for 20 min, the cells were washed with PBS containing 5% FBS. For secondary antibody staining, the cells were stained with streptavidin APC/Cy7, PE/Cy7 anti-mouse IgG1, or APC anti-mouse IgG2a (all from BioLegend, San Diego, CA, USA). Cell death was assessed by annexin V (Corning Inc.) and propidium iodide (PI; Sigma-Aldrich) staining. All samples were analysed on a FACS Canto II (BD Biosciences). The proportion and number of cells relative to the total cell number were analysed using FlowJo software (Tree Star Inc., Ashland, OR, USA).

### RNA extraction and cDNA synthesis

Total RNA was extracted from the total splenocytes, magnetic bead-sorted splenic MRC1^+^ cells or FACS-sorted MRC1^hi^MHCII^lo^ and MRC1^lo^MHCII^hi^ cells by using TRIzol reagent (Invitrogen, Carlsbad, CA, USA) according to the manufacturer’s instructions or an RNeasy kit (Qiagen, Hilden, Germany). Briefly, the cells (5 × 10^5^ cells for FACS-sorted cells and 5 × 10^6^ cells for splenocytes) were treated with 1 mL of TRIzol. Total RNA was isolated by the addition of 200 µL of chloroform followed by centrifugation at 12 000 × *g* for 15 min at 4 °C. The aqueous phase was transferred into a new tube, and 500 µL of isopropanol was added. Then, the samples were incubated for 10 min at room temperature for RNA precipitation and centrifuged at 12 000 × *g* for 10 min at 4 °C. The RNA pellet was obtained after washing with 75% ethanol, air drying for 5–10 min, and resuspension in diethyl pyrocarbonate (DEPC)-treated water. The RNA concentration was quantified using a NanoDrop (Amersham Biosciences, Piscataway, NJ, USA) at 260 nm. Subsequently, 500 ng of purified RNA was reverse-transcribed into cDNA using M-MLV Reverse Transcriptase (Invitrogen) according to the manufacturer’s instructions.

### Quantitative real-time PCR

Quantitative real-time PCR was performed with cDNA using a StepOne Plus real-time PCR system (Applied Biosystems, Foster City, CA, USA). SYBR^®^ Green PCR Master Mix (Applied Biosystems) was used according to the manufacturer’s specifications. The PCR was carried out in a 96-well reaction plate with 10 μL of SYBR^®^ Green PCR Master Mix, 0.5 μL of each of the primers, and 9 μL of cDNA template mixed with DEPC-treated water. Each reaction involved a preincubation at 95 °C for 10 min, followed by 30 thermal cycles at 95 °C for 15 s, 55 °C for 30 s, and 72 °C for 30 s. The relative quantification of the target genes was calculated using the 2^−ΔΔCt^ method. Target gene expression was normalized to the β-actin mRNA level. Primers were designed using NCBI Primer-BLAST as shown in Table 1 and synthesized by Bioneer Inc. (Daejeon, Korea).

**Table 1:** **Primer sequences used for RT-qPCR**

Target gene	Primer sequence (5′–3′)	Accession no.
CCR5	F^*^-GTGGTCAACTGCAAAAAGCA R^#^-GCCCGTTCAACTGTGTCG	NM_001045 834.1
CCR2	F-ATGCCAACAACAACGTTTGA R-TGTTGCCTATGAAGCCAAA	NM_001045835.1
CX3CR1	F-TCCAGAACGATCAAGCACAG R-CGGTGTTCAGTTCCACATTG	XM_418820.2
IL-10	F-CGCTGTCACCGCTTCTTCA R-CGTCTCCTTGATCTGCTTGATG	NM_001004414.2
TGF-β	F-GAGTCCGAGTACTACGCCAAAGA R-CACGTTAAAGCGGAACACATTG	NM_205454.1
COX-2	F-TGCTGGCCGCTCTCCTT R-GTCCTCGTGCAGTCACATTCA	NM_001167719.1
IL-1β	F-ACCCGCTTCATCTTCTACCG R-TCAGCGCCCACTTAGCTTG	NM_204524.1
IL-12p40	F-CCTGTGGCTCGCACTGATAA R-TCTTCGGCAAATGGACAGTA	NM_213571.1
IL-6	F-CGAGTGGGTGCTGTGTCAAA R-CATCCCTGAACGTGTATTTA	XM_015281283.2
iNOS	F-AGCAGCTGAGTGATGATCCA R-GGACCGAGCTGTTGTAGAGA	NM_204961.1

*F: Forward primer for PCR, #R: Reverse primer for PCR.

### Cell sorting

Splenocytes from 3-week-old chickens were stained with mouse anti-chicken MRC1 antibody (clone KUL01) for 20 min at 4 °C. After washing with isolation buffer (PBS containing 0.5% BSA and 2 mM EDTA), the cells were incubated with anti-mouse IgG1 microbeads (Miltenyi Biotec, Bergisch Gladbach, Germany) in isolation buffer for 20 min at 4 °C in the dark. Then, the cells were washed, suspended in isolation buffer, and separated on an LS column in the magnetic field of the MACS Separator (Miltenyi Biotec; > 95% purity). MRC1^hi^MHCII^lo^ and MRC1^lo^MHCII^hi^ cells were sorted with a 70 μm nozzle by using a FACS ARIA III (BD Biosciences) after magnetic bead sorting. The purity of the two subsets was > 99%.

### Phagocytosis assay

For the determination of dead cell uptake, splenocytes from 3-week-old chickens were labelled with CellTrace™ Violet (CTV; Thermo Fisher Scientific) for 10 min and boiled at 56 °C for 30 min to generate dead cells. Cell death was confirmed by annexin V and PI staining. Magnetic bead-sorted splenic MRC1^+^ cells (1 × 10^6^ cell/mL) were cultured in 24-well plates (Thermo Fisher Scientific) with RPMI-1640 supplemented with 10% FBS and 1% penicillin/streptomycin (Invitrogen) at 39 °C in a 5% CO_2_ incubator for 3 h. Then, the dead cells were coincubated with bead-sorted splenic MRC1^+^ cells at a 1:1 ratio for an additional 1 h at either 39 °C or 4 °C. For the determination of ovalbumin (OVA) uptake, bead-sorted MRC1^+^ cells (1 × 10^6^ cell/mL) were cultured for 3 h in a 24-well plate (Thermo Fisher Scientific) followed by 1 h of incubation with OVA-FITC (1–2 μg/mL) at 39 °C or 4 °C. The floating cells and trypsinized adherent cells were collected and washed twice with PBS supplemented with 5% FBS. After staining with anti-chicken MHCII and MRC1, the proportion or intensity of CTV^+^ or OVA-FITC^+^ cells in the MRC1^+^ subsets was examined by using a FACS Canto II. The uptake capability was calculated from the data obtained at 39 °C, which was normalized according to data obtained at 4 °C.

### In vitro migration assay

Migration ability was measured by using a modified Boyden chamber (Corning Costar, Pittsburgh, PA, USA) containing a polycarbonate membrane filter (6.5-mm diameter in a 24-well plate with a 5-μm pore size). The inserts were preincubated with media (RPMI-1640 containing 5% FBS) for 1 h. Magnetic bead-sorted splenic MRC1^+^ cells (1 × 10^6^) were placed in the upper chamber with 200 μL of media, and the lower chamber contained 500 μL media alone or media containing recombinant chicken CCL5 (Kingfisher Biotechnology, Saint Paul, MN, USA). The plates were incubated at 39 °C in 5% CO_2_ for 3 h. The cells that had migrated to the lower chamber were collected, and the cells that remained attached to the insert were recovered by placing the bottom of the insert into 500 μL PBS containing 0.05% (w/v) trypsin–EDTA and tapping it lightly. The migration index was calculated as follows: migration index = [(cell number in the lower chamber with chemokines) − (cell number in the lower chamber without chemokines)]/(number of cells initially added).

### Adoptive transfer assay

To label the injected donor cells, the total PBMCs (1 × 10^7^) and sorted splenic MRC1^lo^MHCII^hi^ cells (5 × 10^6^), isolated from 3-week-old chickens were stained with CTV (Thermo Fisher Scientific) for 10 min at 39 °C in an incubator with 5% CO_2_. The cells were washed with PBS supplemented with 5% FBS and centrifuged at 300–400 × *g* for 3 min at 4 °C. The cells were resuspended in PBS and injected into the 3-week-old recipient chickens through the wing vein. At the same time, to induce systemic inflammation, the chickens were injected with LPS (*Escherichia coli* serotype O111:B4; Sigma-Aldrich) through the intraperitoneal route at a dosage of 1 mg/kg body weight. The control animals received the same amount of PBS.

### Gentamicin protection assay

Phagocytosis and bactericidal activity were examined by a gentamicin protection assay (Additional file 7) [29]. *E. coli* K99 cells were cultured in LB media at 37 °C for 4 h. To prepare the bacterial suspensions, the calculated number (OD_600_ = 1) of *E. coli* K99 cells was centrifuged at 300–400 × *g* for 3 min at 4 °C and resuspended in 6.6 mL RPMI-1640 supplemented with 5% FBS. MRC1^hi^MHCII^lo^ and MRC1^lo^MHCII^hi^ cells (5 × 10^5^ cells/mL) were sorted using a FACS Aria II and incubated at 39 °C in 24-well plates for 3 h. Then, the bacterial suspensions were cocultured with the preincubated MRC1^+^ cell subsets at a multiplicity of infection (MOI) of 20:1 at 39 °C. After 1 h, the cells were washed twice with 1 mL of RPMI-1640 supplemented with 5% FBS, which was replaced with RPMI-1640 containing 200 µg/mL gentamicin (Thermo Fisher Scientific) to kill the remaining extracellular bacteria by incubation for 10 or 60 min. The cells were then washed twice and resuspended in 1 mL of 0.1% Triton X-100 (Sigma-Aldrich) in PBS. To determine the number of colony forming units (CFUs), 100 μL of each serially diluted sample was plated on Tryptic Soy Agar (TSA) (BD Biosciences) followed by overnight incubation at 37 °C.

### Genotyping

Blood samples were collected from randomly chosen White Leghorn chickens. Genomic DNA (gDNA) was extracted from blood by using the AccuPrep^®^ Genomic DNA Extraction Kit (Bioneer Inc.) according to the manufacturer’s instructions. PCRs were performed using 10 ng of genomic DNA, 5 pmol each of the forward and reverse primers, 2 μL of buffer (10x), 2 mM of MgCl_2_, 1 μL of 10 mM dNTPs, 0.25 unit of Taq DNA polymerase (all from Bioneer Inc.) and water in a final volume of 20 μL. The PCR was performed at 94 °C for 5 min, followed by 35 cycles of 94 °C for 30 s, 65 °C for 30 s, and 72 °C for 30 s and a final extension at 72 °C for 5 min. The primers were LEI0258-F: 5′-CACGCAGCAGAACTTGGTAAGG-3′ and LEI0258-R: 5′-AGCTGTGCTC AGTCCTCAGTGC-3′ [30, 31]. BF2 sequence-based typing (SBT) was performed by sequencing each product obtained from the exon 2 region of the chicken BF2 gene using the forward primer 5′-GCAGAGCTCCATACCCTGCGGTA-3′ and the reverse primer 5′-GCCGGTCTGGTTGTAGCGCCG-3′ [32, 33]. All primers were synthesized by Bioneer Inc. PCR products were electrophoresed on a 1.5% agarose gel with 0.5 X TBE buffer. The PCR products were subjected to Sanger sequencing with the forward and reverse primers used for PCR. We retrieved the nucleotide sequence of exon 2 of the chicken MHC class I BF2 gene by using the Ensembl genome browser [34]. Sequence analyses were performed with CLUSTAL Omega [35] and Blast [36].

### Statistical analysis

Statistical analysis (one-way ANOVA with the Tukey post-test or two-way ANOVA with the Bonferroni post-test) was performed using GraphPad Prism (version 7.03, GraphPad Software, San Diego, USA). Differences were considered significant if *P < 0.05, ** P < 0.01, or ***P < 0.001.

## Results

### Two subsets of splenic MRC1^+^ cells showed different expression patterns for MHCII and MRC1

**Figure 1:**
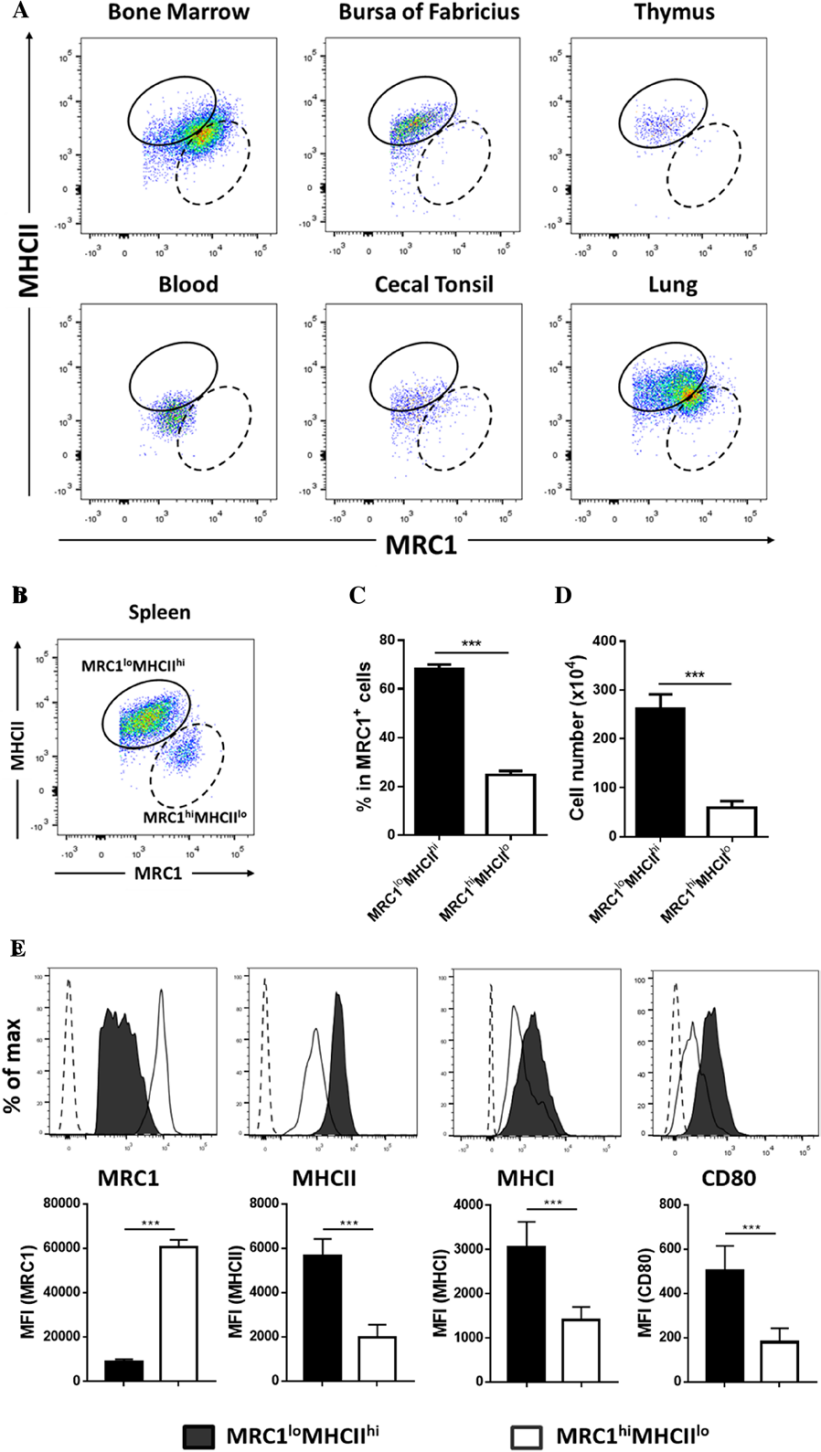
**Two distinct populations of MRC1**^**+**^**cells were found in spleen from chickens based on the expression of MRC1 and MHCII.** Various organs were harvested from 3-week-old chickens. Single cells were stained with anti-chicken MRC1 and MHCII antibodies and analysed by flow cytometry. The gating strategy is shown in Additional file 1. **A** Representative dot plots of MRC1^+^ cells in bone marrow, the bursa of Fabricius, thymus, blood, caecal tonsil, and lung (n = 10). An identical gating strategy for MRC1^lo^MHCII^hi^ and MRC1^hi^MHCII^lo^ cells in the spleen was used in each panel. A representative figure from three independent experiments with similar results is shown. **B** Two distinct populations of spleicn MRC1^+^ cells are shown. The gate with the solid line represents MRC1^lo^MHCII^hi^ cells, and the gate with the dashed line represents MRC1^hi^MHCII^lo^ cells. **C** Proportion and **D** absolute number of MRC1^lo^MHCII^hi^ and MRC1^hi^MHCII^lo^ cells in the spleen. **E** Single cells prepared from the spleen were stained with anti-chicken MRC1, MHCII, MHCI, and CD80 antibodies. Representative histograms display the surface expression of MRC1, MHCI, MHCII or CD80 on MRC1^lo^MHCII^hi^ and MRC1^hi^MHCII^lo^ cell (upper panel). Dashed histograms represent the isotype control. The bar graph represents the mean fluorescence intensity (MFI) of the target molecules (lower panel). Representative histograms are shown with data from three independent experiments with similar results. Data are represented as the mean ± SD. ***P < 0.001.

To investigate the distribution of chicken primary monocyte/macrophage lineage cells in different organs, primary (bone marrow, thymus, and bursa of Fabricius) and secondary (spleen, caecal tonsil, and lung) immune organs from 3-week-old chickens were collected, and their phenotypes were analysed based on the expression of MRC1, a well-known chicken monocyte/macrophage marker (clone: KUL01) [11], and MHCII (Additional file 1). All organs, except for the spleen, showed comparable MRC1 and MHCII levels with homogeneous or indistinguishable expression (Figure 1A). Interestingly, splenic MRC1^+^ cells only showed two distinct populations with heterogeneous expression of MHCII and MRC1. Based on the expression patterns of MRC1 and MHCII, the two populations were designated MRC1^lo^MHCII^hi^ and MRC1^hi^MHCII^lo^ (Figure 1B). In the spleen, the proportion and absolute number of MRC1^lo^MHCII^hi^ cells were higher than those of MRC1^hi^MHCII^lo^ cells at the steady state (Figures 1C and D). MRC1^lo^MHCII^hi^ cells exclusively outnumbered MRC1^hi^MHCII^lo^ cells during the first 3 weeks of life (Additional file 2).

To characterize the phenotypic features of the two MRC1^+^ subsets, cells were stained with several antibodies targeting cell surface markers involved in antigen presentation. The expression of MHCII, MHCI, and CD80 was higher in MRC1^lo^MHCII^hi^ cells than in MRC1^hi^MHCII^lo^ cells (Figure 1E), suggesting that MRC1^lo^MHCII^hi^ cells have better antigen presentation potential than MRC1^hi^MHCII^lo^ cells.

Taken together, these results suggest that, unlike other organs, chicken spleen possesses unique MRC1^+^ populations based on the distinct expression patterns of MRC1 and MHCII.

### Splenic MRC1^hi^MHCII^lo^ cells exhibited greater phagocytic ability than splenic MRC1^lo^MHCII^hi^ cells during steady state conditions

**Figure 2:**
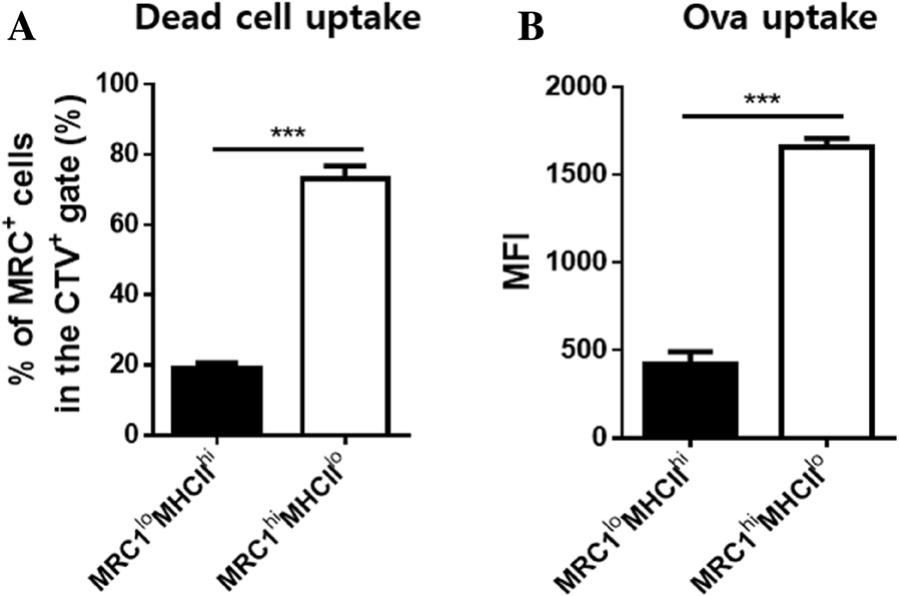
**Splenic MRC1**^**hi**^**MHCII**^**lo**^**cells exhibit phagocytic properties superior to those of MRC1**^**lo**^**MHCII**^**hi**^**cells under steady-state conditions.** For the in vitro phagocytosis assay, MRC1^+^ cells were sorted from splenocytes by positive selection. MRC1^+^ cells (1 × 10^6^ cells/mL) were preincubated for 3 h and then incubated with **A** CTV-labelled dead cells (1 × 10^6^ cells/mL) or **B** OVA-FITC (1 ng/mL) for 1 h at 39 °C or 4 °C. The gating strategy is shown in Additional file 3. Data obtained at 39 °C were normalized according to results obtained at 4 °C. The data represent **A** the percentages of the MRC1^+^ cell subsets in the CTV^+^ gate and **B** the mean fluorescence intensity (MFI) of OVA-FITC in MRC1^lo^MHCII^hi^ and MRC1^hi^MHCII^lo^ cells (*n* = 10). Data are represented as the mean values ± SD. ***P < 0.001.

Phagocytosis of pathogens and aberrant cells is an important task of antigen-presenting cells for the activation of the immune system and the maintenance of homeostasis [1]. To examine the phagocytic activity of MRC1^+^ cells, CTV-labelled dead cells and OVA-FITC were incubated with bead-sorted MRC1^+^ cells. A higher number of CTV^+^ cells was detected among MRC1^hi^MHCII^lo^ cells than among MRC1^lo^MHCII^hi^ cells (Figure 2A and Additional file 3A), indicating the increased uptake of dead cells. Similarly, OVA uptake was also much higher in MRC1^hi^MHCII^lo^ cells than in MRC1^lo^MHCII^hi^ cells (Figure 2B and Additional file 3B). These results suggest that MRC1^hi^MHCII^lo^ cells have superior phagocytosis properties compared to those of MRC1^lo^MHCII^hi^ cells.

### Splenic MRC1^hi^MHCII^lo^ cells showed higher migration capacity than splenic MRC1^lo^MHCII^hi^ cells

**Figure 3:**
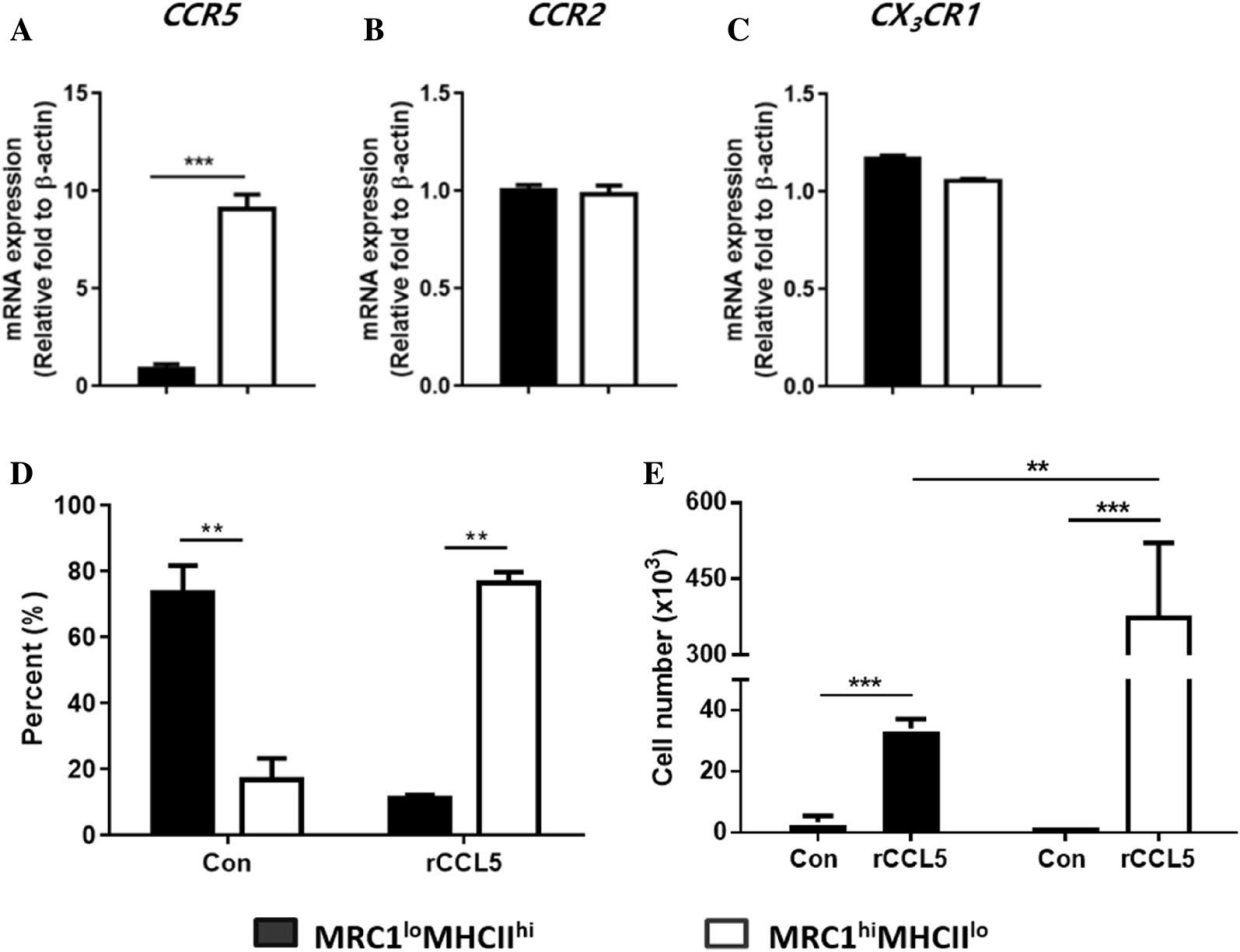
**Splenic MRC1**^**hi**^**MHCII**^**lo**^**cells migrated towards CCL5 better than MRC1**^**lo**^**MHCII**^**hi**^**cells.A-C** Chicken splenocytes were sorted into MRC1^lo^MHCII^hi^ and MRC1^hi^MHCII^lo^ cell populations using a cell sorter. The transcriptional levels of the chicken chemokine receptors **A** CCR5, **B** CCR2 and **C** CX_3_CR1 were analysed by RT-qPCR. Their expression was normalized to the β-actin mRNA level (*n* = 10). Data are represented as the mean values ± SD. ***P < 0.001. **D**, **E** For the in vitro migration assay, bead-sorted splenic MRC1^+^ cells (1 × 10^6^ cells/mL) in 200 μL of media were placed in the insert of the transwell plate. The lower compartment was filled with media in the absence or presence of recombinant chicken CCL5 protein (50 ng/mL). After 1 h, **D** the percentage and **E** absolute number of migrated cells in the lower compartment were quantified by flow cytometry (*n* = 10). Data are represented as the mean values ± SD. **P < 0.01 ***P < 0.001.

Monocytes/macrophages possess migration capacity, which is important in regulating infection and inflammation [5, 37] and in maintaining organ homeostasis, even in the steady state [38, 39]. To evaluate the migration capacity, the two subsets were examined for their transcriptional expression of chemokine receptors. The transcription level of CCR5 was significantly upregulated in MRC1^hi^MHCII^lo^ cells compared to that in MRC1^lo^MHCII^hi^ cells (Figure 3A). However, comparable expression levels of CCR2 and CX_3_CR1 were found in both MRC1^+^ subsets (Figures 3B and C). Next, a migration assay was performed on bead-sorted MRC1^+^ cells using recombinant chicken CCL5, a ligand of CCR5. As shown in Figure 1C, splenic MRC1^+^ cells contained a high proportion of MRC1^lo^MHCII^hi^ cells (approximately 70%) and a low proportion of MRC1^hi^MHCII^lo^ cells (approximately 30%). After migration in the absence of CCL5 (i.e., spontaneous migration), MRC1^lo^MHCII^hi^ cells were detected at high levels in the bottom well. However, a high percentage and number of MRC1^hi^MHCII^lo^ cells were observed (Figures 3D and E) when CCL5 was added, which is consistent with high CCR5 expression on MRC1^hi^MHCII^lo^ cells. We confirmed that 3 h of culture with or without CCL5 did not induce the alteration of MHCII and MRC1 expression in the migration assay (data not shown). Taken together, the results indicated that MRC1^hi^MHCII^lo^ cells showed a CCR5-dependent migratory pattern.

### Inflammation caused an increase in both the proportion and absolute number of splenic MRC1^hi^MHCII^lo^ cells

**Figure 4:**
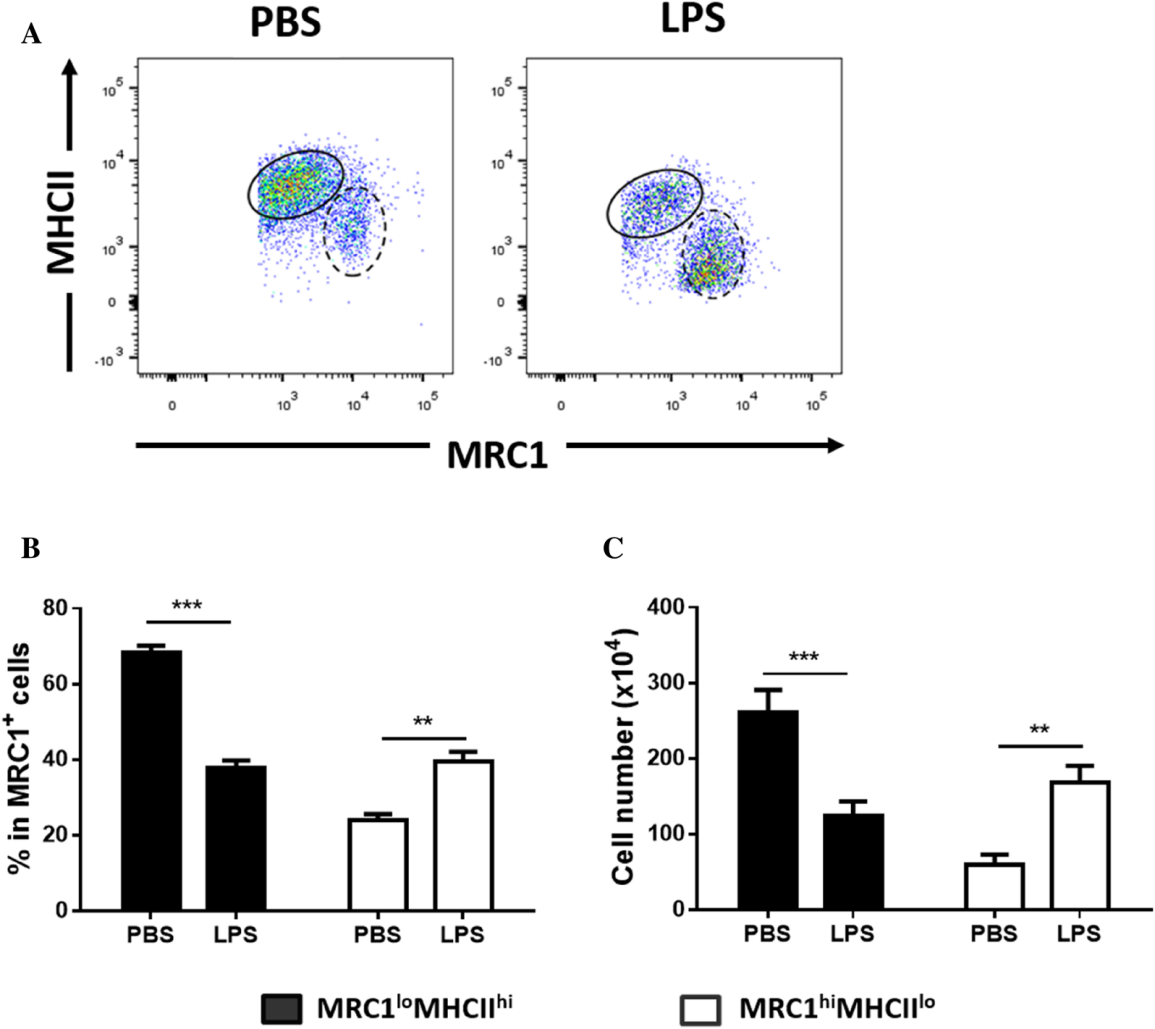
**LPS-induced inflammation in chickens resulted in changes in the proportion and number of the two populations of splenic MRC1**^**+**^**cells.** Chickens were administered PBS or LPS (1 mg/kg body weight) i.p. After 4 h, the spleens were collected, and the splenocytes were analysed to determine the number and proportional changes of the subtypes of MRC1^+^ cells by flow cytometry. **A** Representative dot plot with similar results showing MRC1^+^ cells from the spleen of chickens administered PBS or LPS. The solid line represents MRC1^lo^MHCII^hi^ cells, whereas the dashed line represents MRC1^hi^MHCII^lo^ cells. The bar graph represents the **B** proportion and **C** absolute number of the two subsets in the PBS or LPS administration group (*n* = 10). Data are represented as the mean values ± SD. **P < 0.01, ***P < 0.001.

Next, to further characterize splenic MRC1^+^ cells under inflammatory conditions in vivo, chickens were treated with LPS through the intraperitoneal route. No obvious differences were found in the composition of immune cells, including MRC1^+^ cells, B cells, and T cells, in the spleen at 4 h after stimulation (Additional file 4). While two MRC1^+^ subpopulations were detected, their proportion and absolute number were greatly altered in spleens from LPS-treated chickens (Figure 4A). MRC1^lo^MHCII^hi^ cells were the major population (over 65%) among splenic MRC1^+^ cells in the PBS-treated control group, which was similar to that found in the spleen in the steady state (Figure 1B). However, at 4 h post-LPS stimulation, the proportion and absolute number of MRC1^hi^MHCII^lo^ cells were greatly increased, resulting in a reversal of the ratio of the two populations (Figures 4B, C). Collectively, the results showed that LPS-induced inflammation caused a change in the proportion and absolute number of the subsets of MRC1^+^ cells.

### Inflammation triggered phenotypic changes in splenic MRC1^+^ cells

**Figure 5:**
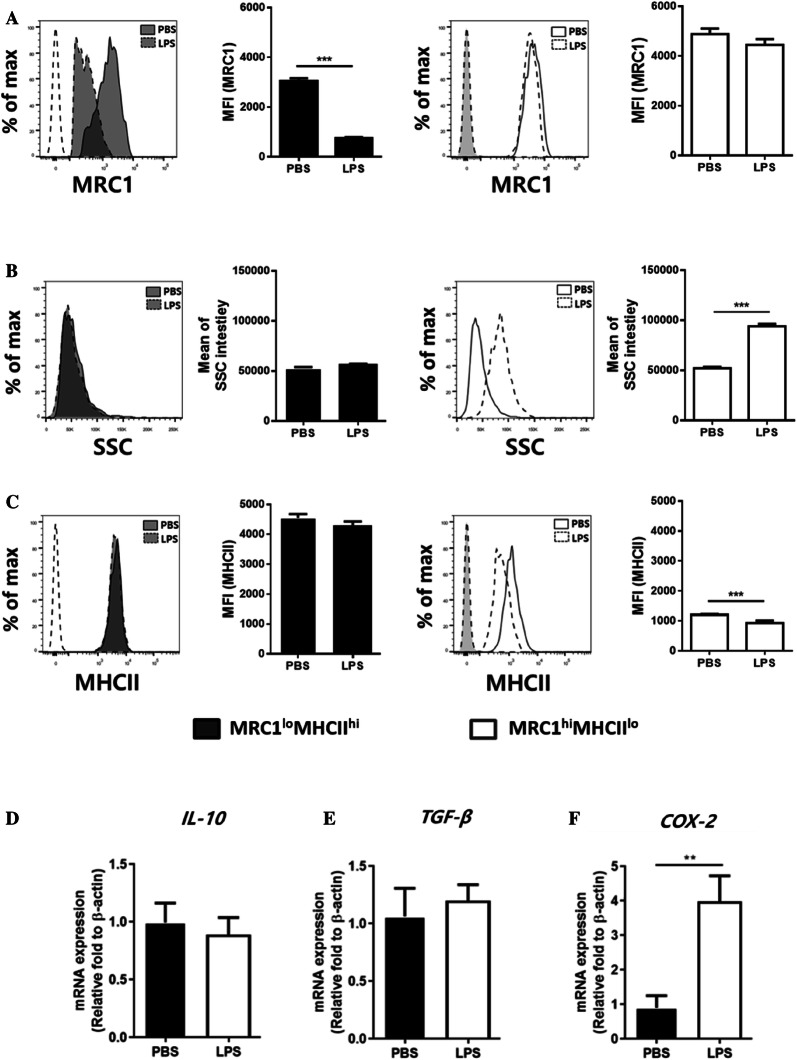
**LPS-induced inflammation in chickens decreased MHCII expression in splenic MRC1**^**hi**^**MHCII**^**lo**^**cells.** Chickens were administered PBS or LPS (500 μL of 1 mg/kg body weight) i.p. for 4 h. Then, the spleen was collected and analysed to determine the **A-C** phenotype and **D-F** mRNA levels of potential mediators through flow cytometry and RT-qPCR, respectively. Representative histograms show the changes in **A** MRC1, **B** SSC, and **C** MHCII expression in the two subsets at 4 h after LPS injection. Solid histograms represent the PBS group, and dashed histograms show the LPS group. Dashed histograms without colour (left) and with colour (right) represent the isotype control. The bar graph represents the MFI of the expression (*n* = 10) **P < 0.01, ***P < 0.001. Transcriptional levels of **D** IL-10, **E** TGF-β, and **F** COX-2 in splenocytes were examined by RT-qPCR. The expression was normalized to the β-actin mRNA level (*n* = 10). Data are represented as the mean values ± SD. **P < 0.01.

In mammals, LPS can trigger phenotypic changes by activating mononuclear phagocytes both in vivo and in vitro [40]. Chicken macrophage cell lines treated with LPS showed a similar response [41]. In the present study, the phenotypes of the two splenic MRC1^+^ subpopulations were analysed in chickens that had been administered with LPS via the intraperitoneal route. The expression level of MRC1 was further decreased in MRC1^lo^MHCII^hi^ cells but not in MRC1^hi^MHCII^lo^ cells (Figure 5A). However, granularity (indicated by side scatter, SSC) was greatly increased in MRC1^hi^MHCII^lo^ cells showing a reduction in MHCII expression at 4 h after LPS administration (Figures 5B and C).

A few factors have been known to reduce the expression of MHCII, including TGF-β [42], IL-10 [43], and COX-2 [44, 45]. To elucidate the potential mediators that negatively regulate MHCII expression in the spleen during inflammation, the transcription levels of TGF-β, IL-10, and COX-2 in total splenocytes during inflammation were analysed by RT-qPCR. The mRNA expression levels of IL-10 and TGF-β were similar between the PBS and LPS groups (Figures 5D and E). Interestingly, however, the level of COX-2 was greatly elevated in the LPS group compared to that in the PBS group (Figure 5F), suggesting that COX-2 might be a potential mediator responsible for the downregulation of MHCII expression.

### Two subsets of splenic MRC1^+^ cells showed distinct phenotypic changes during LPS-induced inflammation

**Figure 6:**
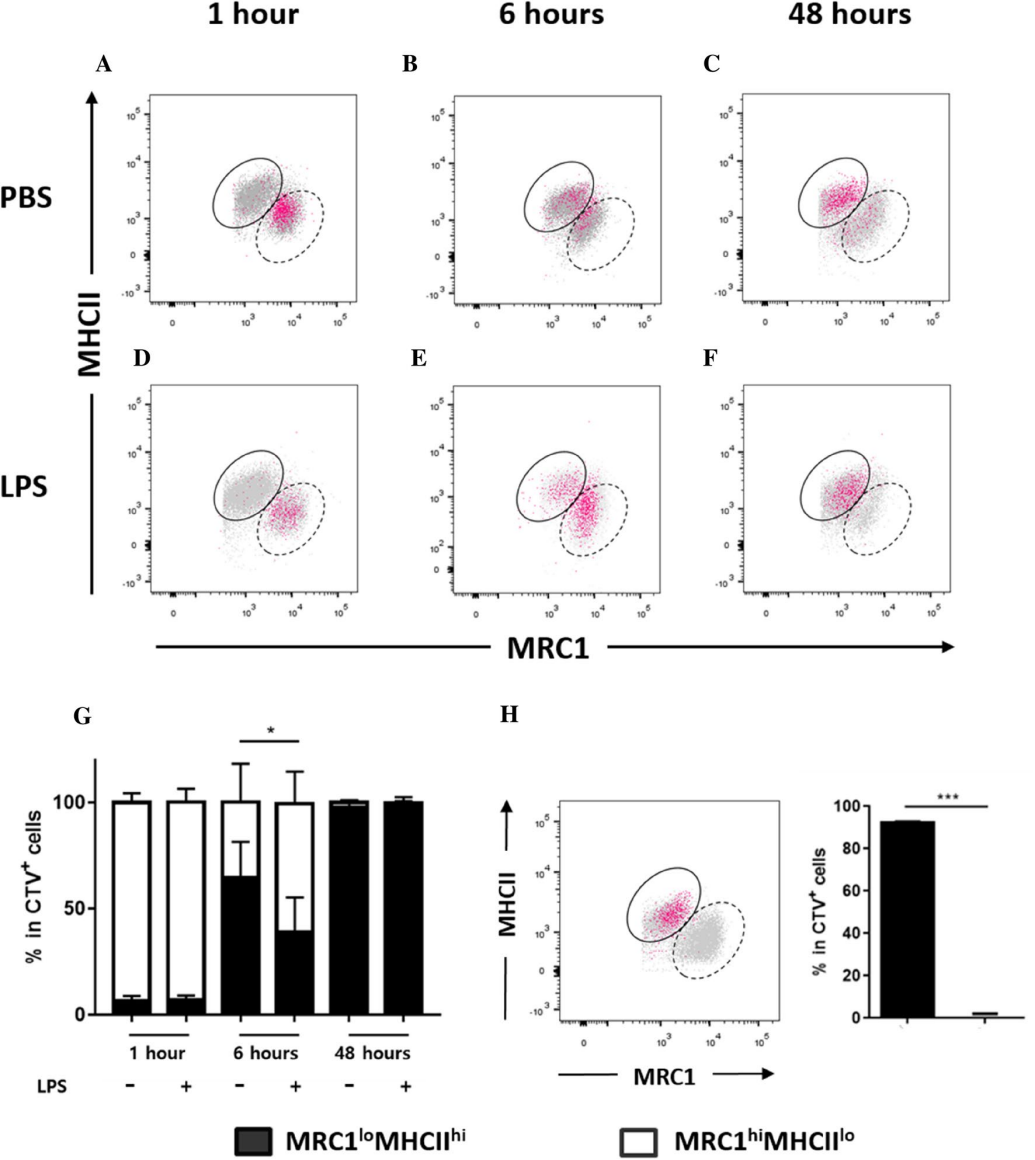
**Splenic MRC1**^**+**^**cells were replenished by monocytes and showed distinct phenotypic changes in chickens with LPS-induced inflammation. A**–**G** PBMCs were isolated from 3-week-old chickens, labelled with CTV, and transferred into age-matched recipient chickens via the intravenous route (200 μL of 1 × 10^7^ cells). To cause LPS-induced inflammation, recipient chickens were intraperitoneally administered 200 μL of LPS (1 mg/kg body weight) or the same volume of PBS (control) immediately after the cell transfer. At 1, 6, and 48 h after LPS administration, spleens were isolated from recipient chickens, and the splenocytes were stained with anti-chicken MRC1 and MHCII antibodies. Injected PBMCs were distinguished from recipient cells (grey dots) by analysing the CTV^+^-pre-gated cells (pink dots). In addition, the MRC1^+^ cells were gated, and the expression of MRC1 and MHCII was examined by flow cytometry for MRC1^lo^MHCII^hi^ (solid line) and MRC1^hi^MHCII^lo^ cells (dashed line). Representative dot plot of the results from 1-, 6-, and 48-h post-adoptive transfer **A**–**F** showed the proportion of donor MRC1^+^ cells (pink) within the recipient MRC1^+^ populations (grey) in the spleen. The bar graph **G** represents the proportion of MRC1^lo^MHCII^hi^ and MRC1^hi^MHCII^lo^ cells within CTV^+^MRC1^+^ donor cells in the recipient after adoptive transfer (*n* = 10). **H** MRC1^lo^MHCII^hi^ cells were sorted from the spleen of 3-week-old chickens and labelled with CTV. CTV-labelled MRC1^lo^MHCII^hi^ cells were transferred into age-matched recipient chickens via the intravenous route (200 μL of 1 × 10^7^ cells). Then, the recipient chickens were intraperitoneally administered 200 μL of LPS (1 mg/kg body weight). Pink dots represent donor cells, and grey dots recipient cells. The bar graph displays the proportions of MRC1^lo^MHCII^hi^ and MRC1^hi^MHCII^lo^ cells within CTV^+^MRC1^+^ cells (pink in the left panel) at 6 h after the cell transfer (*n* = 6). Data are represented as the mean values ± SD. * *P* < 0.05, *** *P* < 0.001.

In mammals, tissue resident macrophages are seeded in the prenatal stage and maintain their population by self-renewal [46] and/or are replenished by the infiltration of monocytes [38, 47]. To elucidate whether the two subsets of splenic MRC1^+^ cells were replenished by circulating monocytes, we performed cell transfer using blood or splenic cells from chickens that maintained conserved haplotypes in the MHC region (Additional file 5). PBMCs were isolated from blood, labelled with CTV, and transferred into recipient chickens. Then, the kinetic changes in the two splenic MRC1^+^ subsets were analysed in CTV-positive cells from recipient animals. Circulating MRC1^+^ cells from PBMCs exhibited the homogenous expression of MRC1 and MHCII in both control and LPS-treated chickens (Additional file 6). At 1 h post-transfer, most CTV-positive cells were detected in the MRC1^hi^MHCII^lo^ population (Figures 6A and G), indicating that MRC1^hi^MHCII^lo^ cells represent newly infiltrated cells from blood. However, CTV-positive cells were found and gradually increased in number within the MRC1^lo^MHCII^hi^ population at 6 and 48 h after cell transfer (Figures 6B, C and G). Collectively, the results showed that the two splenic MRC1^+^ subsets were replenished by monocytes and MRC1^lo^MHCII^hi^ cells were derived from MRC1^hi^MHCII^lo^ cells.

To examine how MRC1^hi^MHCII^lo^ cells were increased during LPS-induced inflammation (Figures 4B and C), we hypothesized two possibilities: i) a delay in the transformation of MRC1^hi^MHCII^lo^ cells into MRC1^lo^MHCII^hi^ cells and ii) phenotype change of MRC1^lo^MHCII^hi^ cells into MRC1^hi^MHCII^lo^ cells.

Thus, CTV-labelled PBMCs were adoptively transferred via the wing vein in age-matched recipients, and LPS injection was performed through the intraperitoneal route at the same time. At 1 h post-adoptive transfer, CTV-labelled cells were readily observed within the MRC1^hi^MHCII^lo^ population in the LPS-treated groups (Figures 6D and G), similar to that observed in the control (Figures 6A and G). However, at 6 h post-adoptive transfer, CTV-positive cells were still observed at high numbers within the MRC1^hi^MHCII^lo^ population in the LPS-treated group (Figures 6E and G) compared to that in the PBS control (Figures 6B and G), indicating that LPS-induced inflammation delayed the appearance of donor cells in the MRC1^lo^MHCII^hi^ cell population gate. Notably, in the LPS-treated group at 48 h post-adoptive transfer, most CTV^+^ cells were found within the MRC1^lo^MHCII^hi^ cell population (Figures 6F and G), which was similar to that observed in the PBS group at 48 h post-adoptive transfer (Figures 6C and G).

Another possible explanation for the increase in MRC1^hi^MHCII^lo^ cells during LPS-induced inflammation could be the phenotypic change of MRC1^lo^MHCII^hi^ cells into MRC1^hi^MHCII^lo^ cells. To investigate whether MRC1^lo^MHCII^hi^ cells changed their phenotype during inflammation, CTV-labelled MRC1^lo^MHCII^hi^ cells were adoptively transferred via the wing vein, and i.p. LPS injection was performed at the same time. At 6 h post-adoptive transfer, almost all CTV-positive cells were found within the MRC1^lo^MHCII^hi^ gate (Figure 6H), suggesting that MRC1^lo^MHCII^hi^ cells did not change their phenotype in LPS-induced inflammation conditions. Collectively, the results indicated that the increase in MRC1^hi^MHCII^lo^ cells during inflammation was caused by a delay in the transformation of MRC1^hi^MHCII^lo^ cells into MRC1^lo^MHCII^hi^ cells.

### Splenic MRC1^lo^MHCII^hi^ cells were more likely to show cytokine expression compared to splenic MRC1^hi^MHCII^lo^ cells

**Figure 7:**
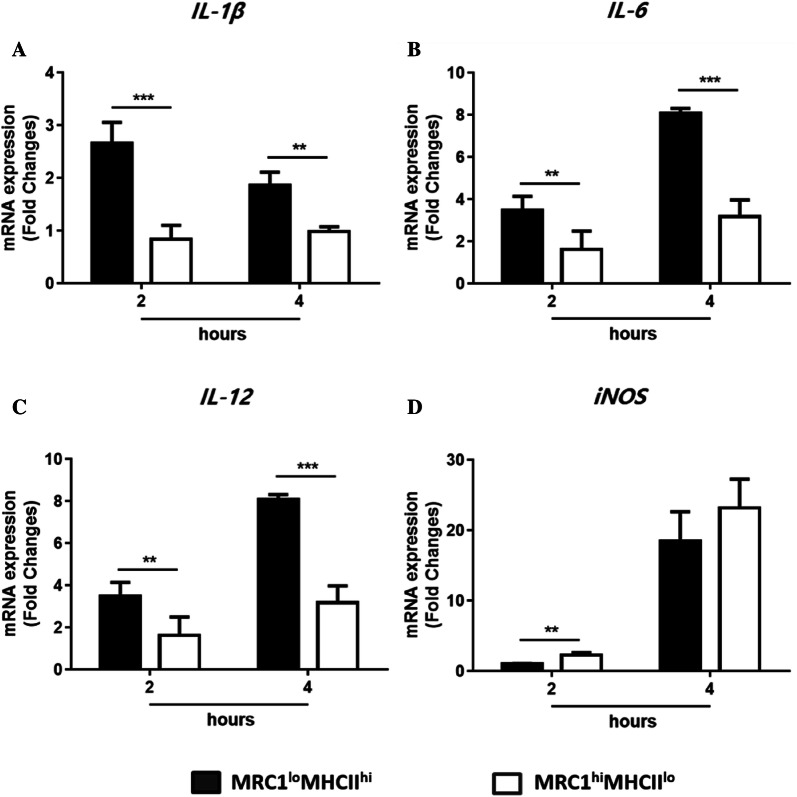
**Splenic MRC1**^**lo**^**MHCII**^**hi**^**cells were the main source of inflammatory cytokines.** The mRNA expression of **A** IL-1β, **B** IL-6, **C** IL-12, and **D** iNOS in FACS-sorted MRC1^lo^MHCII^hi^ and MRC1^hi^MHCII^lo^ cells from the spleen of chickens at 2 and 4 h post-LPS injection. Data are normalized according to the data of the PBS group (*n* = 10). Data are represented as the mean values ± SD. **P < 0.01, ***P < 0.001.

Monocytes/macrophages are the major source of inflammatory mediators during inflammation [8]. To investigate their ability to produce inflammatory mediators, the two splenic cell subsets were sorted from spleen after LPS administration, and the mRNA expression of IL-1β, IL-6, IL-12p40, and iNOS were examined. The transcription levels of pro-inflammatory cytokines, such as IL-1β, IL-6 and IL-12p40, were higher in MRC1^lo^MHCII^hi^ cells than in MRC1^hi^MHCII^lo^ cells at both 2 and 4 h post-LPS injection (Figures 7A–C). No significant difference was found in iNOS mRNA expression at 4 h post-LPS injection, although iNOS mRNA expression in MRC1^hi^MHCII^lo^ cells was higher than that in MRC1^lo^MHCII^hi^ cells at 2 h post-LPS injection (Figure 7D). Together, the results showed that MRC1^lo^MHCII^hi^ cells have a greater capability to express inflammatory cytokines than MRC1^hi^MHCII^lo^ cells.

### Splenic MRC1^hi^MHCII^lo^ cells showed greater bactericidal activity than splenic MRC1^lo^MHCII^hi^ cells during LPS-induced inflammation

**Figure 8:**
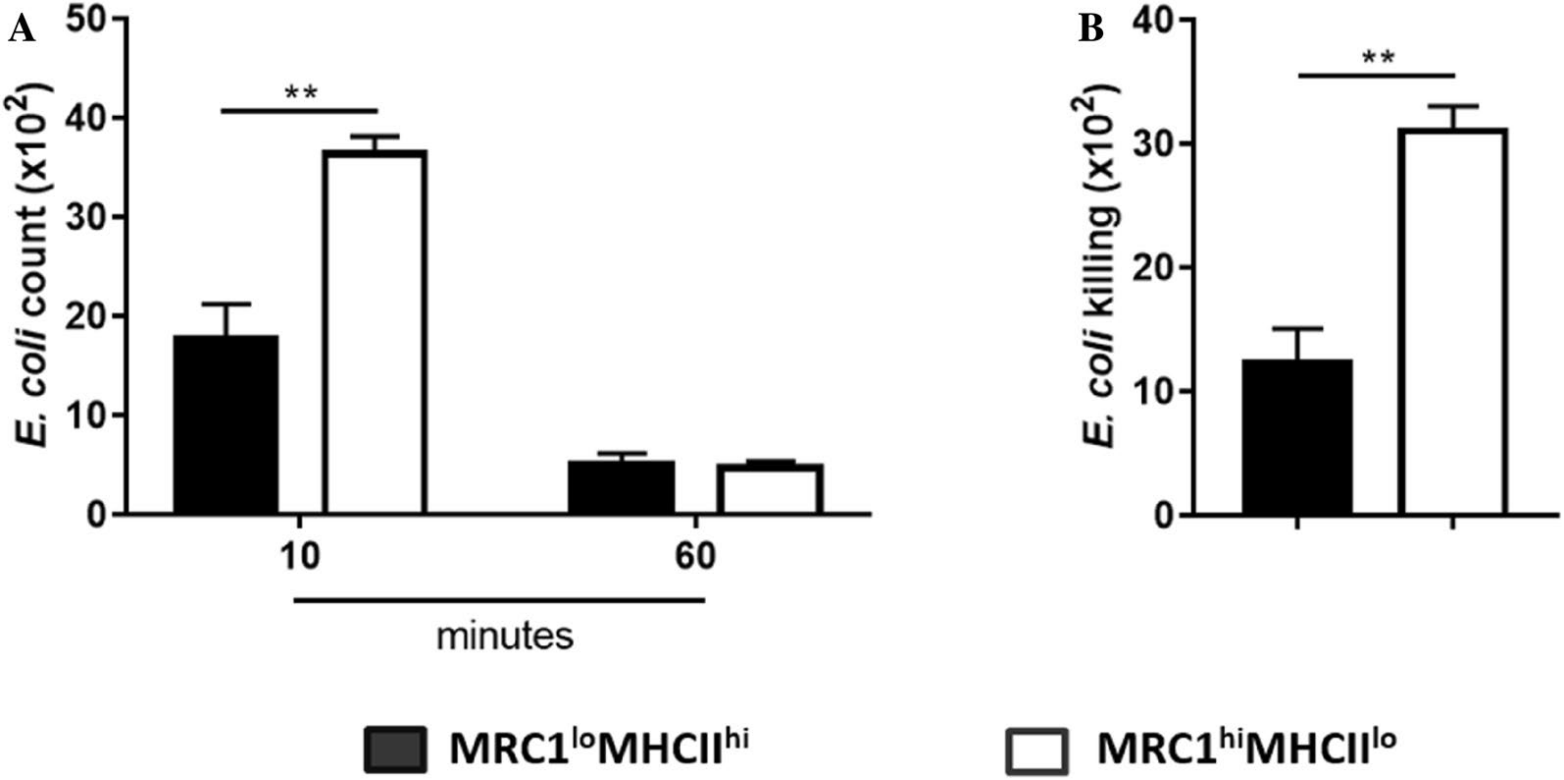
**Splenic MRC1**^**hi**^**MHCII**^**lo**^**cells showed a superior bacterial killing capacity compared to that of MRC1**^**lo**^**MHCII**^**hi**^**cells when treated with LPS. A** The bar graph represents the remaining bacteria in the FACS-sorted MRC1^lo^MHCII^hi^ and MRC1^hi^MHCII^lo^ cell lysates after 10 (uptake assay) or 60 min (clearance assay) of gentamicin treatment. **B** The bar graph represents the bacterial killing capacity of MRC1^lo^MHCII^hi^ and MRC1^hi^MHCII^lo^ cells. Data were calculated by subtracting the number of bacteria remaining after clearance from the number from the uptake assay (*n* = 10). Data are represented as the mean values ± SD. **P < 0.01.

The results showed that LPS-induced inflammation increased the proportion and absolute number of MRC1^hi^MHCII^lo^ cells (Figures 4B and C), while the main source of inflammatory cytokines during inflammation was MRC1^lo^MHCII^hi^ cells. Interestingly, however, granularity, as indicated by SSC (Figure 5B) and the mRNA expression of iNOS (Figure 7D), were significantly increased in MRC1^hi^MHCII^lo^ cells after LPS administration. Therefore, a gentamicin protection assay was conducted to evaluate the bactericidal activity of the cells (Additional file 7). Since the uptake capability of the two subsets was different in steady state conditions (Figures 2B and C), gentamicin was added to the cells 10 or 60 min prior to the test of bacterial uptake or clearance, respectively. The results showed similar rates of bacterial clearance in both cell lysates; however, bacterial uptake was higher in MRC1^hi^MHCII^lo^ cells than in MRC1^lo^MHCII^hi^ cells (Figure 8A). Thus, similar to that observed in steady-state conditions, MRC1^hi^MHCII^lo^ cells are more likely to exhibit bacterial uptake compared to MRC1^lo^MHCII^hi^ cells during LPS-induced inflammation. Interestingly, however, MRC1^hi^MHCII^lo^ cells have greater bactericidal activity than MRC1^lo^MHCII^hi^ cells (Figure 8B). Taken together, the results indicated that the increase in MRC1^hi^MHCII^lo^ cells during LPS-induced inflammation is more likely to be responsible for the increase in bactericidal activity than the increase in MRC1^lo^MHCII^hi^ cells.

## Discussion

In chickens, very few studies have been conducted on the function of primary mononuclear phagocytes. CD11c^+^ and MRC1^+^ cells have been reported to be analogous to mammalian DCs and monocyte/macrophage lineage cells, respectively [13]. Although the presence of these two subpopulations has also been reported among splenic MRC1^+^ cells, the functional characteristics of splenic MRC1^+^ cells have not been fully elucidated. Therefore, in the present study, splenic MRC1^+^ subpopulations were characterized into two subsets based on their surface expression of MHCII and MRC1 and were examined for their phenotype, function, and response to inflammatory stimulation (Table 2).

**Table 2:** **Phenotypic and functional characteristics of splenic MRC1**^**lo**^**MHCII**^**hi**^**and MRC1**^**hi**^**MHCII**^**lo**^**monocyte/macrophage lineage cells during steady state and inflammatory conditions**

Characteristics	MRC1^lo^MHCII^hi^	MRC1^hi^MHCII^lo^
Phenotype	High MHCII, MHCI, CD80	High MRC1
Macrophages signature genes*	**↑↑**	**↑**
Phagocytosis	**↑**	**↑↑**
Migration	**↑**	**↑↑**
Proportion	Decreased	Increased
Phenotype change	Decreased MRC1	Increased granularity Decreased MHCII
Cytokines	**↑↑**	**↑**
Bactericidal activity	**↑**	**↑↑**

The last four lines represent the characteristics of splenic MRC1^lo^MHCII^hi^ and MRC1^hi^MHCII^lo^ cells during LPS-induced inflammation.

Phagocytosis of dead cells and pathogens is one of the most important functional features of tissue resident macrophages in maintaining the homeostasis of tissues and organs [3]. In the present study, MRC1^hi^MHCII^lo^ cells showed higher phagocytic activity than MRC1^lo^MHCII^hi^ cells. The exact mechanism involved in the phagocytosis of apoptotic cells and pathogens has yet to be fully elucidated. Several receptors, such as T cell membrane protein 4 (TIM4) [48], receptor tyrosine kinase Mer (MerTK) [49], and mannose receptor [50], are known for their important role in the recognition and phagocytosis of apoptotic cells and pathogens in mice. Similarly, the role of TIM4 in phagocytosing apoptotic cells has been reported in chickens [18]. Therefore, the higher phagocytic activity of MRC1^hi^MHCII^lo^ cells compared to that of MRC1^lo^MHCII^hi^ cells is likely linked to the expression level of MRC1, a chicken mannose receptor [14].

Macrophages are functionally classified as M1 and M2 types in mice and humans [51, 52]. M1 macrophages express the well-known signature markers iNOS and CD80 and show the upregulation of MHCII, and they exhibit pro-inflammatory responses that often function to clear invading pathogens. M2 macrophages, which express CD206 and PPAR-γ as signature markers, play a role in immunoregulation and tissue repair, including the phagocytosis of dead cells. However, the exact definition and functional relevance of M1 and M2 macrophages in chickens has not been established. In the present study, MRC1^hi^MHCII^lo^ cells phagocytosed dead cells better and expressed higher levels of MRC1, which is homologous to the mammalian mannose receptor (CD206), than MRC1^lo^MHCII^hi^ cells, suggesting that MRC1^hi^MHCII^lo^ cells may act as M2-like macrophages in chickens. Functional studies of MRC1^hi^MHCII^lo^ cells as M2 macrophages should be conducted to refine the M1/M2 macrophage dichotomy in chickens.

Compared to MRC1^hi^MHCII^lo^ cells, MRC1^lo^MHCII^hi^ cells showed a high capability of expressing pro-inflammatory cytokines (IL-1β, IL-6, and IL-12) during LPS-induced inflammation in the present study, suggesting that MRC1^lo^MHCII^hi^ cells are more likely to show cytokine expression than MRC1^hi^MHCII^lo^ cells. MRC1^hi^MHCII^lo^ cells show a low level of pro-inflammatory cytokine expression, which appears to be closely related to their phagocytic activity (Figure 2B), as the uptake of apoptotic bodies repressed the production of inflammatory mediators [53]. Indeed, red pulp macrophages with phagocytic activity against apoptotic cells in mice showed low IL-1β mRNA expression [54]. Therefore, MRC1^lo^MHCII^hi^ cells clearly expressed cytokines, whereas the uptake of apoptotic cells was greater in MRC1^hi^MHCII^lo^ cells than in MRC1^hi^MHCII^lo^ cells. However, since the expression of cytokines and other inflammatory mediators were evaluated only at the genetic level in the present study, the expression of cytokines and other inflammatory mediators should also be examined at the protein level to improve understanding.

The adoptive cell transfer technique has been widely used to demonstrate the origin [38, 55] and function [56, 57] of mononuclear phagocytes in mouse studies, whereas very few studies have been conducted in chickens. To the best of our knowledge, this is the first study in chickens demonstrating the replenishment of tissue resident macrophages in conjunction with the monocytic infiltration of PBMCs. Adoptive transfer of CTV-labelled PBMCs via the intravenous route in chickens demonstrated that the observed MRC1^hi^MHCII^lo^ cells were newly infiltrated into spleen, in which their phenotype switched to that of MRC1^lo^MHCII^hi^ cells at later time points. Unlike mammalian tissue resident macrophages, which are commonly self-maintained with a reduced monocyte contribution [47], the present data suggest that splenic monocytes/macrophages could be replenished by monocyte infiltration in chickens. However, the absolute number of MRC1^lo^MHCII^hi^ cells was also decreased at 4 h post-LPS administration, suggesting the possibility of the migration of MRC1^lo^MHCII^hi^ cells into the inflammatory site (i.e., peritoneal cavity). Therefore, further study on the migration of splenic MRC1^+^ cells to other sites, such as the peritoneum, could demonstrate where and how MRC1^+^ cells react to inflammatory stimuli.

Similar to that observed in mammalian spleen, two kinds of macrophages (red pulp macrophages and ellipsoid-associated macrophages) are observed in chicken spleen [11]. However, the exact location of MRC1^lo^MHCII^hi^ and MRC1^hi^MHCII^lo^ cells in the spleen is yet to be determined. Therefore, in future studies, immunohistochemical or live imaging approaches should be used to demonstrate the location and timing of the phenotypic changes, if any, in the two subsets.

MHCII, which is mainly expressed on antigen presenting cells, is considered an activation marker together with the costimulatory molecules (CD86 and CD80) in DCs and macrophages. For example, when bone marrow-derived immature DCs and M0 macrophages are treated with LPS or IFN-γ, the surface expression of MHCII and costimulatory molecules is elevated, and immature cells are differentiated into activated mature DCs and M1 macrophages, respectively [40]. Furthermore, treatment with several TLR ligands also induced the upregulation of MHCII expression along with an increase in the expression of co-stimulatory molecules in chicken macrophage cell lines [41]. However, in the present study, in contrast to the expectation that LPS injection would induce the elevation of MHCII expression, LPS-induced inflammation caused a decrease in MHCII expression on MRC1^hi^MHCII^lo^ cells. Interestingly, however, no significant difference was observed in MRC1^lo^MHCII^hi^ cells.

It is well known in mammals that the transcription of MHCII is tightly regulated by the master coactivator class II MHC transactivator (CIITA) [58]. The exact mechanism of MHCII downregulation during LPS-induced inflammation was not elucidated in the current study, although the factors known to regulate MHCII expression were measured in spleen treated with LPS administered i.p. It has been reported that in mammals, COX-2, which is increased under inflammatory conditions, can reduce MHCII expression by regulating CIITA via prostaglandin-dependent cAMP production [59]. In the present study, COX-2 showed a remarkable elevation in transcription in spleen that was treated with LPS i.p., suggesting that COX-2 may be a potential MHCII regulator during LPS-induced inflammation. Given that COX-2 can be produced in almost all types of immune cells during inflammatory conditions [60], the exact source and location of COX-2 production were not determined in the present study. Thus, it is hard to determine why MRC1^hi^MHCII^lo^ cells were more affected by the increase in COX-2 in the spleen than MRC1^lo^MHCII^hi^ cells.

In conclusion, the current study demonstrated that chicken splenocytes contain phenotypically and functionally distinct subsets of monocyte/macrophage lineage cells. Moreover, MRC1^lo^MHCII^hi^ cells displayed a tendency to express inflammatory cytokines, whereas MRC1^hi^MHCII^lo^ cells showed elevated mannose receptor expression and phagocytosis activity, implying that the two subsets might play distinct roles during infection (for instance, in antigen presentation and pathogen clearance, respectively). Further studies are necessary to elucidate the precise role of the subsets in protective immunity against extracellular and intracellular pathogens. Collectively, the results of the present study provide insight to improve the understanding of the splenic monocyte/macrophage lineage cells of the avian immune system.

## Supplementary information

**Additional file 1. Flow cytometry gating strategy and isotype controls used for MRC1**^**+**^**cells.A** Gating strategy used for MRC1^lo^MHCII^hi^ and MRC1^hi^MHCII^lo^ cells. Single cells were gated based on size and granularity using FSC-A and SSC-A and then sub-gated using 7-AAD to define the live cells. Then, the live cells were discriminated by the expression of MRC1-PE (clone: KUL01), and then the MRC1^lo^MHCII^hi^ (solid line) and MRC1^hi^MHCII^lo^ (broken line) cells were defined based on the expression of MRC1 and MHCII. **B** The background level of fluorescence was examined using the proper isotype controls.

**Additional file 2. General features of splenic MRC1**^**+**^**cells.A** Proportion and **B** absolute number of splenic mononuclear phagocytes from 1- to 3-week-old chickens (*n* = 10). Data are represented as the mean values ± SD. ***P <0.01.

**Additional file 3. Gating strategy used for the dead cell and OVA uptake assays.A** Splenocytes from 3-week-old chickens were labelled with CTV for 10 min and boiled at 56 °C for 30 min to inactivate the cells. Dead cells were co-incubated with bead-sorted splenic MRC1^+^ cells at a 1:1 ratio for 1 h. Then, dead cell uptake was determined according to the CTV^+^ cells among the MRC1^lo^MHCII^hi^ and MRC1^hi^MHCII^lo^ cells using flow cytometry. **B** OVA uptake was determined by analysing intensity of FITC in MRC1^lo^MHCII^hi^ and MRC1^hi^MHCII^lo^ cells. Then, the uptake ability was calculated from the data obtained at 39 °C, which was normalized according to data obtained at 4 °C. The histogram presents the intensity of FITC in each subset.

**Additional file 4. Influence of i.p. injection of LPS into chickens.** Percentage and absolute number of splenic leukocytes after 4 **h of treatment with PBS or LPS (*n* = 10).** Data are represented as the mean values ± SD.

**Additional file 5. Genotyping of White Leghorn chickens based on LEI0258 and BF2 exon 2.** The gDNA was extracted from blood samples of four randomly selected White Leghorn Chickens at the University Animal Farm. LEI0258- and BF2-targeting PCRs were performed. Additionally, BF2 SBT was performed by sequencing the BF2 exon 2 region from each of the four chickens. PCR amplification of the MHC-B haplotypes using **A** LEI0258 microsatellites and **B** BF2 exon 2. **C** Nucleotide sequence alignment for BF2 exon 2 sequence-based typing. M; DNA marker (100 bp), S1-4; gDNA samples from each of the 4 chickens.

**Additional file 6. Blood MRC1**^**+**^**monocytes showed the homogenous expression of MRC1 and MHCII.** Blood was collected and diluted in PBS at a 1:1 ratio. PBMCs, which were isolated from diluted blood by using Ficoll-Paque, were stained with anti-MRC1 and MHCII antibodies and analysed by using flow cytometry. Representative dot plot of MRC1^+^ monocytes from the PBMCs of chickens administered PBS or LPS.

**Additional file 7. Schematic diagram of the gentamicin protection assay.** MRC1^lo^MHCII^hi^ (filled) and MRC1^hi^MHCII^lo^ (open) cells were sorted by a FACS Aria II and coincubated with *E. coli* K99 for 1 h. After co-incubation, gentamicin (200 μg/mL) was used to kill the extracellular bacteria for 10 min (uptake assay) or 60 min (clearance assay). Cell lysates were incubated overnight on TSA agar to measure the CFU value.

## Data Availability

The data sets used and analysed during the current study are available from the corresponding author upon reasonable request.

## References

[1] van Furth R, Cohn ZA, Hirsch JG, Humphrey JH, Spector WG, Langevoort HL (1972). The mononuclear phagocyte system: a new classification of macrophages, monocytes, and their precursor cells. Bull World Health Organ.

[2] Gomez Perdiguero E, Klapproth K, Schulz C, Busch K, Azzoni E, Crozet L, Garner H, Trouillet C, de Bruijn MF, Geissmann F, Rodewald HR (2015). Tissue-resident macrophages originate from yolk-sac-derived erythro-myeloid progenitors. Nature.

[3] Li MO, Sarkisian MR, Mehal WZ, Rakic P, Flavell RA (2003). Phosphatidylserine receptor is required for clearance of apoptotic cells. Science.

[4] Munoz-Espin D, Canamero M, Maraver A, Gomez-Lopez G, Contreras J, Murillo-Cuesta S, Rodriguez-Baeza A, Varela-Nieto I, Ruberte J, Collado M, Serrano M (2013). Programmed cell senescence during mammalian embryonic development. Cell.

[5] Shi C, Pamer EG (2011). Monocyte recruitment during infection and inflammation. Nat Rev Immunol.

[6] Gordon S (2002). Pattern recognition receptors: doubling up for the innate immune response. Cell.

[7] Ip WK, Sokolovska A, Charriere GM, Boyer L, Dejardin S, Cappillino MP, Yantosca LM, Takahashi K, Moore KJ, Lacy-Hulbert A, Stuart LM (2010). Phagocytosis and phagosome acidification are required for pathogen processing and MyD88-dependent responses to Staphylococcus aureus. J Immunol.

[8] Arango Duque G, Descoteaux A (2014). Macrophage cytokines: involvement in immunity and infectious diseases. Front Immunol.

[9] Borges da Silva H, Fonseca R, Pereira RM, Cassado Ados A, Alvarez JM, D’Imperio Lima MR (2015). Splenic Macrophage Subsets and Their Function during Blood-Borne Infections. Front Immunol.

[10] John JL (1994). The avian spleen: a neglected organ. Q Rev Biol.

[11] Mast J, Goddeeris BM, Peeters K, Vandesande F, Berghman LR (1998). Characterisation of chicken monocytes, macrophages and interdigitating cells by the monoclonal antibody KUL01. Vet Immunol Immunopathol.

[12] Quéré P, Pierre J, Hoang MD, Esnault E, Domenech J, Sibille P, Dimier-Poisson I (2013). Presence of dendritic cells in chicken spleen cell preparations and their functional interaction with the parasite *Toxoplasma gondii*. Vet Immunol Immunopathol.

[13] Vu Manh TP, Marty H, Sibille P, Le Vern Y, Kaspers B, Dalod M, Schwartz-Cornil I, Quéré P (2014). Existence of conventional dendritic cells in *Gallus gallus* revealed by comparative gene expression profiling. J Immunol.

[14] Staines K, Hunt LG, Young JR, Butter C (2014). Evolution of an expanded mannose receptor gene family. PLoS One.

[15] Sabet T, Wen-Cheng H, Stanisz M, El-Domeiri A, Van Alten P (1977). A simple method for obtaining peritoneal macrophages from chickens. J Immunol Methods.

[16] Carrel A, Ebeling AH (1922). Pure cultures of large mononuclear leucocytes. J Exp Med.

[17] Myszewski MA, Stern NJ (1991). Phagocytosis and intracellular killing of *Campylobacter jejuni* by elicited chicken peritoneal macrophages. Avian Dis.

[18] Hu T, Wu Z, Bush SJ, Freem L, Vervelde L, Summers KM, Hume DA, Balic A, Kaiser P (2019). Characterization of subpopulations of chicken mononuclear phagocytes that express TIM4 and CSF1R. J Immunol.

[19] Wigley P (2013). Immunity to bacterial infection in the chicken. Dev Comp Immunol.

[20] Coorens M, van Dijk A, Bikker F, Veldhuizen EJ, Haagsman HP (2015). Importance of endosomal cathelicidin degradation to enhance DNA-induced chicken macrophage activation. J Immunol.

[21] Xie H, Raybourne RB, Babu US, Lillehoj HS, Heckert RA (2003). CpG-induced immunomodulation and intracellular bacterial killing in a chicken macrophage cell line. Dev Comp Immunol.

[22] Iqbal M, Philbin VJ, Smith AL (2005). Expression patterns of chicken Toll-like receptor mRNA in tissues, immune cell subsets and cell lines. Vet Immunol Immunopathol.

[23] Barjesteh N, Behboudi S, Brisbin JT, Villanueva AI, Nagy E, Sharif S (2014). TLR ligands induce antiviral responses in chicken macrophages. PLoS One.

[24] Wigley P, Hulme S, Rothwell L, Bumstead N, Kaiser P, Barrow P (2006). Macrophages isolated from chickens genetically resistant or susceptible to systemic salmonellosis show magnitudinal and temporal differential expression of cytokines and chemokines following *Salmonella enterica* challenge. Infect Immun.

[25] Oláh I, Nagy N, Vervelde L, Schat KA, Kaspers B, Kaiser P (2014). Chapter 2-Structure of the Avian Lymphoid System. Avian Immunology.

[26] Lee IK, Gu MJ, Ko KH, Bae S, Kim G, Jin GD, Kim EB, Kong YY, Park TS, Park BC, Jung HJ, Han SH, Yun CH (2018). Regulation of CD4(+)CD8(-)CD25(+) and CD4(+)CD8(+)CD25(+) T cells by gut microbiota in chicken. Sci Rep.

[27] Ko KH, Lee IK, Kim G, Gu MJ, Kim HY, Park BC, Park TS, Han SH, Yun CH (2018). Changes in bursal B cells in chicken during embryonic development and early life after hatching. Sci Rep.

[28] Autissier P, Soulas C, Burdo TH, Williams KC (2010). Evaluation of a 12-color flow cytometry panel to study lymphocyte, monocyte, and dendritic cell subsets in humans. Cytometry A.

[29] Wu J, Pugh R, Laughlin RC, Andrews-Polymenis H, McClelland M, Baumler AJ, Adams LG (2014). High-throughput assay to phenotype *Salmonella enterica* Typhimurium association, invasion, and replication in macrophages. J Vis Exp..

[30] Fulton JE, Juul-Madsen HR, Ashwell CM, McCarron AM, Arthur JA, O’Sullivan NP, Taylor RL (2006). Molecular genotype identification of the *Gallus gallus* major histocompatibility complex. Immunogenetics.

[31] Tu Y, Shu J, Ji G, Zhang M, Zou J (2018). Monitoring conservation effects on a Chinese indigenous chicken breed using major histocompatibility complex B-G gene and DNA Barcodes. Asian-Australas J Anim Sci.

[32] Livant EJ, Ewald SJ (2005). High-resolution typing for chicken BF2 (MHC class I) alleles by automated sequencing. Anim Genet.

[33] Seo JH, Lee JH, Kong HS (2017). Assessment of genetic diversity and phylogenetic relationships of Korean native chicken breeds using microsatellite markers. Asian-Australas J Anim Sci.

[34] BF2-201 ENSGALT00000079478. https://asia.ensembl.org/Gallus_gallus/Transcript/Exons?db=core;g=ENSGALG00000041380;r=16:2606528-2609057;t=ENSGALT00000079478. Accessed 27 Dec 2019

[35] CLUSTAL Omega. https://www.ebi.ac.uk/Tools/msa/clustalo/

[36] Blast. https://blast.ncbi.nlm.nih.gov/Blast.cgi

[37] Vogel DY, Heijnen PD, Breur M, de Vries HE, Tool AT, Amor S, Dijkstra CD (2014). Macrophages migrate in an activation-dependent manner to chemokines involved in neuroinflammation. J Neuroinflammation.

[38] Tamoutounour S, Guilliams M, Montanana Sanchis F, Liu H, Terhorst D, Malosse C, Pollet E, Ardouin L, Luche H, Sanchez C, Dalod M, Malissen B, Henri S (2013). Origins and functional specialization of macrophages and of conventional and monocyte-derived dendritic cells in mouse skin. Immunity.

[39] Bain CC, Bravo-Blas A, Scott CL, Perdiguero EG, Geissmann F, Henri S, Malissen B, Osborne LC, Artis D, Mowat AM (2014). Constant replenishment from circulating monocytes maintains the macrophage pool in the intestine of adult mice. Nat Immunol.

[40] Labeur MS, Roters B, Pers B, Mehling A, Luger TA, Schwarz T, Grabbe S (1999). Generation of tumor immunity by bone marrow-derived dendritic cells correlates with dendritic cell maturation stage. J Immunol.

[41] Sunkara LT, Achanta M, Schreiber NB, Bommineni YR, Dai G, Jiang W, Lamont S, Lillehoj HS, Beker A, Teeter RG, Zhang G (2011). Butyrate enhances disease resistance of chickens by inducing antimicrobial host defense peptide gene expression. PLoS ONE.

[42] Romieu-Mourez R, Francois M, Boivin MN, Stagg J, Galipeau J (2007). Regulation of MHC class II expression and antigen processing in murine and human mesenchymal stromal cells by IFN-gamma, TGF-beta, and cell density. J Immunol.

[43] Lee KS, Baek DW, Kim KH, Shin BS, Lee DH, Kim JW, Hong YS, Bae YS, Kwak JY (2005). IL-10-dependent down-regulation of MHC class II expression level on monocytes by peritoneal fluid from endometriosis patients. Int Immunopharmacol.

[44] Harizi H, Juzan M, Pitard V, Moreau JF, Gualde N (2002). Cyclooxygenase-2-issued prostaglandin e(2) enhances the production of endogenous IL-10, which down-regulates dendritic cell functions. J Immunol.

[45] Harizi H, Grosset C, Gualde N (2003). Prostaglandin E2 modulates dendritic cell function via EP2 and EP4 receptor subtypes. J Leukoc Biol.

[46] Yona S, Kim KW, Wolf Y, Mildner A, Varol D, Breker M, Strauss-Ayali D, Viukov S, Guilliams M, Misharin A, Hume DA, Perlman H, Malissen B, Zelzer E, Jung S (2013). Fate mapping reveals origins and dynamics of monocytes and tissue macrophages under homeostasis. Immunity.

[47] Hashimoto D, Chow A, Noizat C, Teo P, Beasley MB, Leboeuf M, Becker CD, See P, Price J, Lucas D, Greter M, Mortha A, Boyer SW, Forsberg EC, Tanaka M, van Rooijen N, Garcia-Sastre A, Stanley ER, Ginhoux F, Frenette PS, Merad M (2013). Tissue-resident macrophages self-maintain locally throughout adult life with minimal contribution from circulating monocytes. Immunity.

[48] Miyanishi M, Tada K, Koike M, Uchiyama Y, Kitamura T, Nagata S (2007). Identification of Tim4 as a phosphatidylserine receptor. Nature.

[49] Scott RS, McMahon EJ, Pop SM, Reap EA, Caricchio R, Cohen PL, Earp HS, Matsushima GK (2001). Phagocytosis and clearance of apoptotic cells is mediated by MER. Nature.

[50] Garcia-Aguilar T, Espinosa-Cueto P, Magallanes-Puebla A, Mancilla R (2016). The mannose receptor is involved in the phagocytosis of Mycobacteria-induced apoptotic cells. J Immunol Res.

[51] Italiani P, Boraschi D (2014). From monocytes to M1/M2 macrophages: phenotypical vs functional differentiation. Front Immunol.

[52] Martinez FO, Gordon S (2014). The M1 and M2 paradigm of macrophage activation: time for reassessment. F1000 Prime Rep.

[53] Roberts AW, Lee BL, Deguine J, John S, Shlomchik MJ, Barton GM (2017). Tissue-resident macrophages are locally programmed for silent clearance of apoptotic cells. Immunity.

[54] Ag N, Quintana JA, Garcia-Silva S, Mazariegos M, Gonzalez de la Aleja A, Nicolas-Avila JA, Walter W, Adrover JM, Crainiciuc G, Kuchroo VK, Rothlin CV, Peinado H, Castrillo A, Ricote M, Hidalgo A (2017). Phagocytosis imprints heterogeneity in tissue-resident macrophages. J Exp Med.

[55] Zigmond E, Jung S (2013). Intestinal macrophages: well educated exceptions from the rule. Trends Immunol.

[56] Ji J, Shu D, Zheng M, Wang J, Luo C, Wang Y, Guo F, Zou X, Lv X, Li Y, Liu T, Qu H (2016). Microbial metabolite butyrate facilitates M2 macrophage polarization and function. Sci Rep.

[57] Parsa R, Andresen P, Gillett A, Mia S, Zhang XM, Mayans S, Holmberg D, Harris RA (2012). Adoptive transfer of immunomodulatory M2 macrophages prevents type 1 diabetes in NOD mice. Diabetes.

[58] Harton JA, Ting JP (2000). Class II transactivator: mastering the art of major histocompatibility complex expression. Mol Cell Biol.

[59] Li G, Harton JA, Zhu X, Ting JP (2001). Downregulation of CIITA function by protein kinase a (PKA)-mediated phosphorylation: mechanism of prostaglandin E, cyclic AMP, and PKA inhibition of class II major histocompatibility complex expression in monocytic lines. Mol Cell Biol.

[60] Caughey GE, Cleland LG, Penglis PS, Gamble JR, James MJ (2001). Roles of cyclooxygenase (COX)-1 and COX-2 in prostanoid production by human endothelial cells: selective up-regulation of prostacyclin synthesis by COX-2. J Immunol.

