# Supplementary figures and images for: Characterization of splenic MRC1hiMHCIIlo and MRC1loMHCIIhi cells from the monocyte/macrophage lineage of White Leghorn chickens
Source: Vet Res. 2020 May 27;51:73. doi: 10.1186/s13567-020-00795-9 (PMC7251834; doi:10.1186/s13567-020-00795-9)

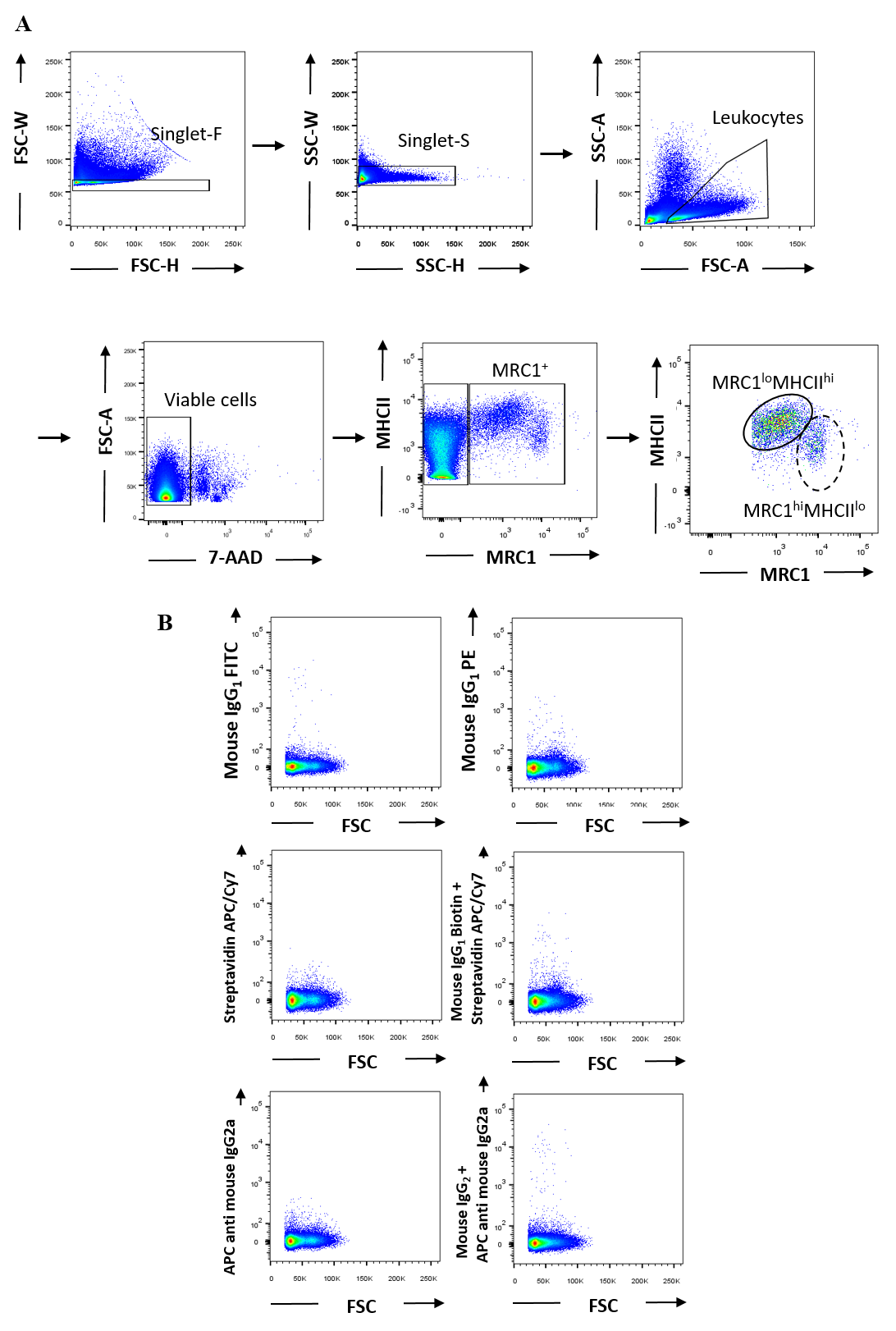

Supplement: Supplementary file 1 — Additional file 1. Flow cytometry gating strategy and isotype controls used for MRC1+cells.A Gating strategy used for MRC1loMHCIIhi and MRC1hiMHCIIlo cells. Single cells were gated based on size and granularity using FSC-A and SSC-A and then sub-gated using 7-AAD to define the live cells. Then, the live cells were discriminated by the expression of MRC1-PE (clone: KUL01), and then the MRC1loMHCIIhi (solid line) and MRC1hiMHCIIlo (broken line) cells were defined based on the expression of MRC1 and MHCII. B The background level of fluorescence was examined using the proper isotype controls. [file 13567_2020_795_MOESM1_ESM.docx]

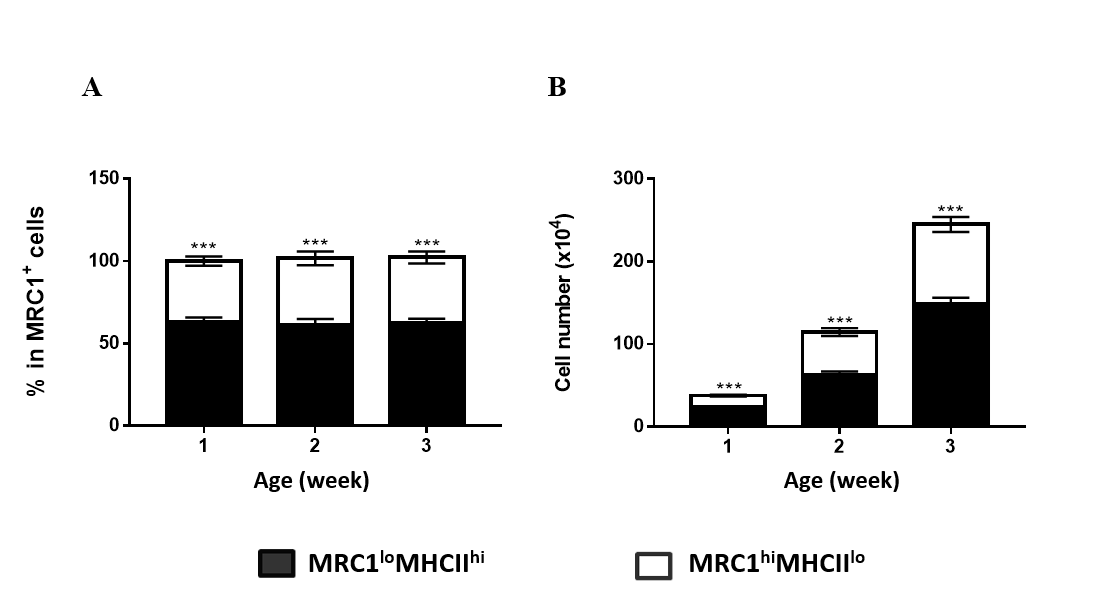

Supplement: Supplementary file 2 — Additional file 2. General features of splenic MRC1+cells.A Proportion and B absolute number of splenic mononuclear phagocytes from 1- to 3-week-old chickens (n = 10). Data are represented as the mean values ± SD. ***P <0.01. [file 13567_2020_795_MOESM2_ESM.docx]

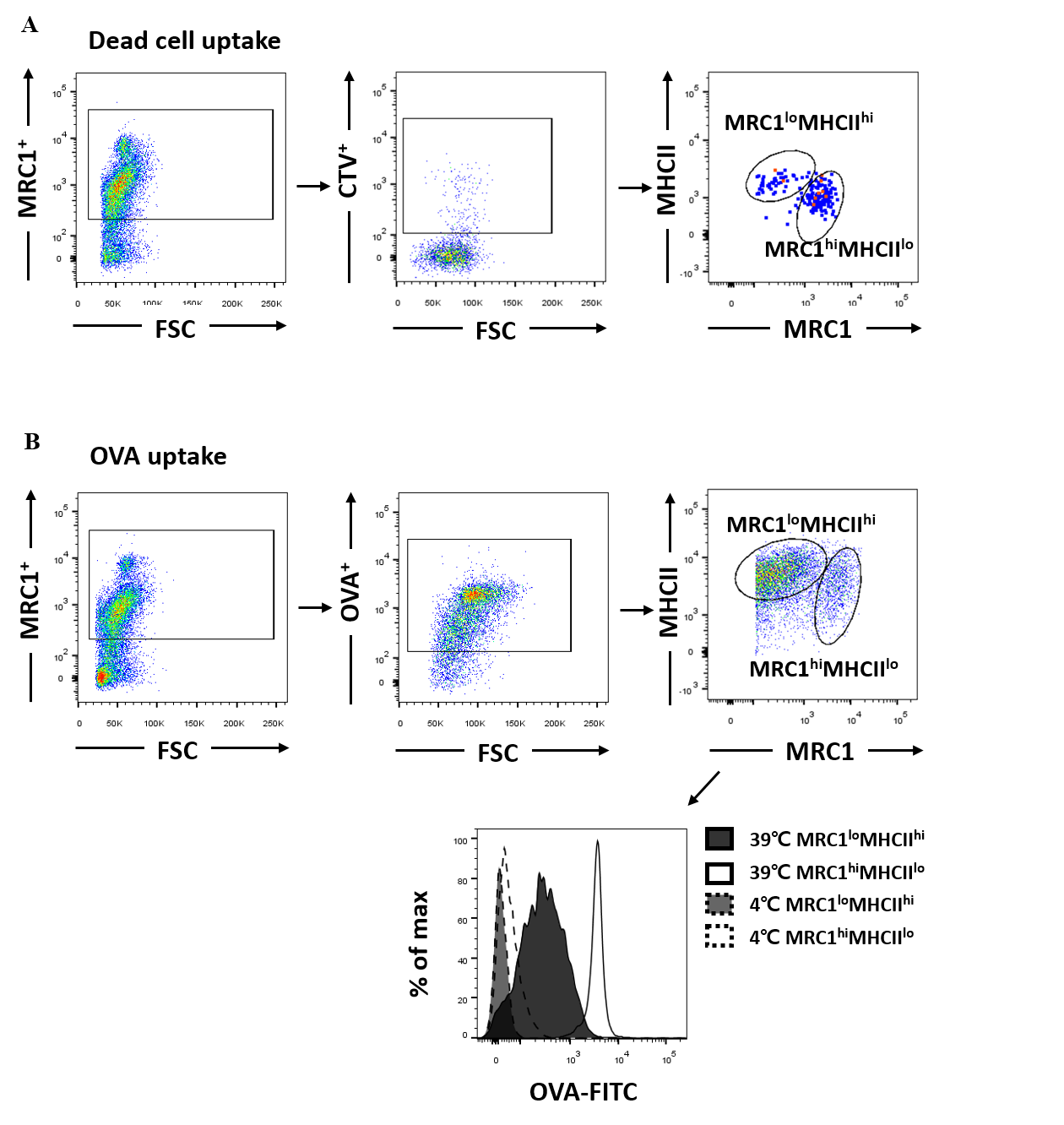

Supplement: Supplementary file 3 — Additional file 3. Gating strategy used for the dead cell and OVA uptake assays.A Splenocytes from 3-week-old chickens were labelled with CTV for 10 min and boiled at 56 °C for 30 min to inactivate the cells. Dead cells were co-incubated with bead-sorted splenic MRC1+ cells at a 1:1 ratio for 1 h. Then, dead cell uptake was determined according to the CTV+ cells among the MRC1loMHCIIhi and MRC1hiMHCIIlo cells using flow cytometry. B OVA uptake was determined by analysing intensity of FITC in MRC1loMHCIIhi and MRC1hiMHCIIlo cells. Then, the uptake ability was calculated from the data obtained at 39 °C, which was normalized according to data obtained at 4 °C. The histogram presents the intensity of FITC in each subset. [file 13567_2020_795_MOESM3_ESM.docx]

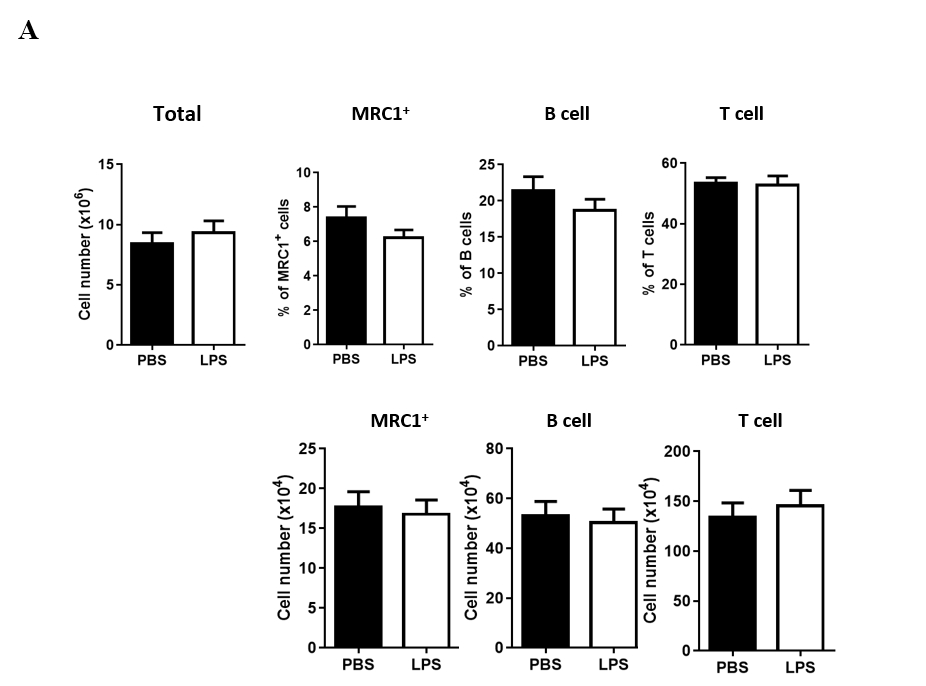

Supplement: Supplementary file 4 — Additional file 4. Influence of i.p. injection of LPS into chickens. Percentage and absolute number of splenic leukocytes after 4 h of treatment with PBS or LPS (n = 10). Data are represented as the mean values ± SD. [file 13567_2020_795_MOESM4_ESM.docx]

**
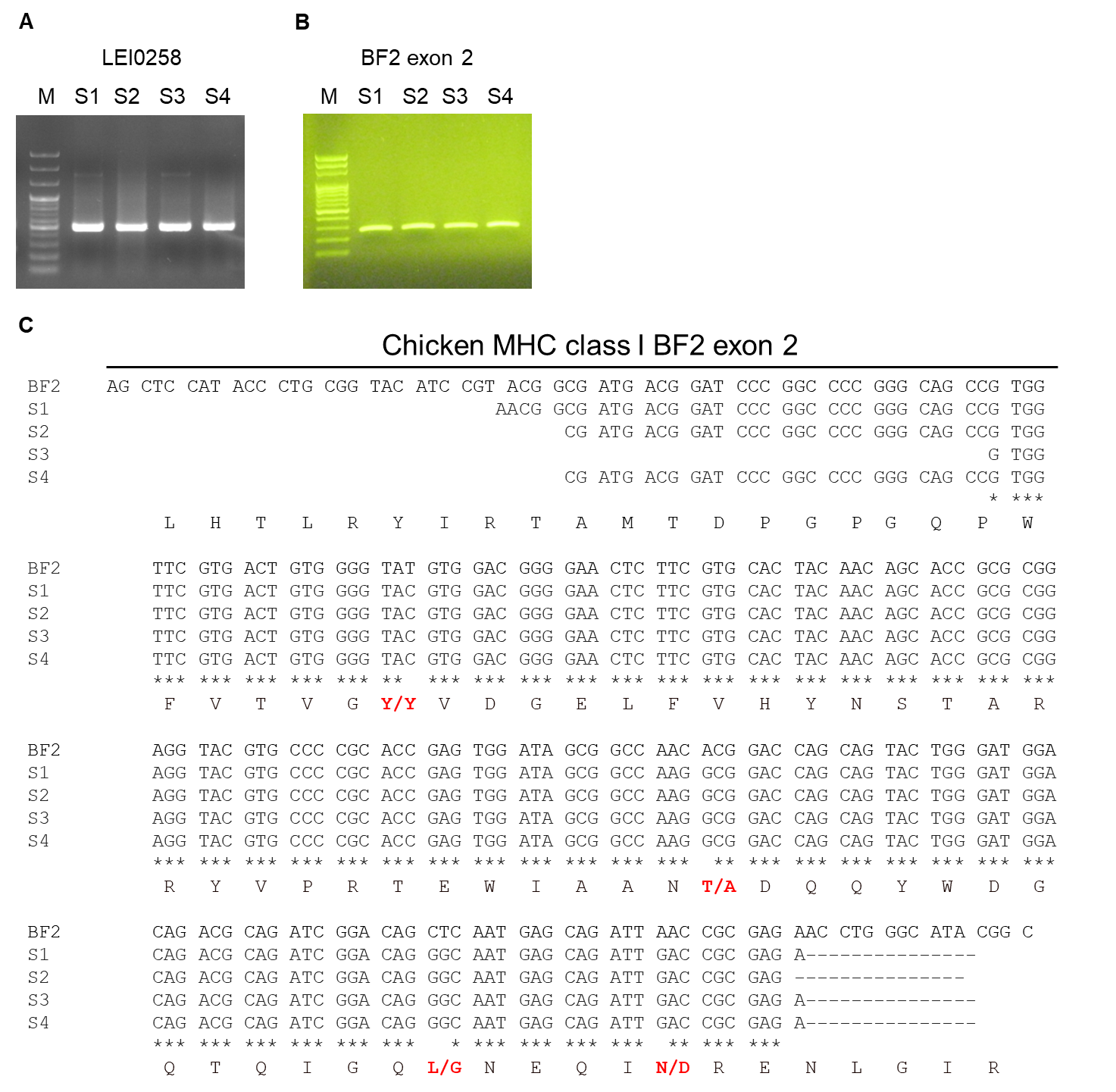
**

Supplement: Supplementary file 5 — Additional file 5. Genotyping of White Leghorn chickens based on LEI0258 and BF2 exon 2. The gDNA was extracted from blood samples of four randomly selected White Leghorn Chickens at the University Animal Farm. LEI0258- and BF2-targeting PCRs were performed. Additionally, BF2 SBT was performed by sequencing the BF2 exon 2 region from each of the four chickens. PCR amplification of the MHC-B haplotypes using A LEI0258 microsatellites and B BF2 exon 2. C Nucleotide sequence alignment for BF2 exon 2 sequence-based typing. M; DNA marker (100 bp), S1-4; gDNA samples from each of the 4 chickens. [file 13567_2020_795_MOESM5_ESM.docx]

**
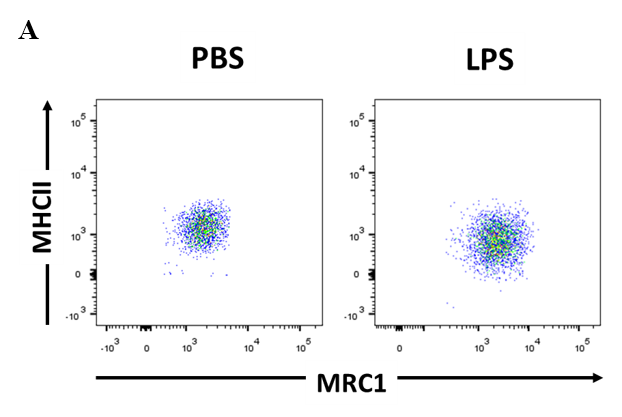
**

Supplement: Supplementary file 6 — Additional file 6. Blood MRC1+monocytes showed the homogenous expression of MRC1 and MHCII. Blood was collected and diluted in PBS at a 1:1 ratio. PBMCs, which were isolated from diluted blood by using Ficoll-Paque, were stained with anti-MRC1 and MHCII antibodies and analysed by using flow cytometry. Representative dot plot of MRC1+ monocytes from the PBMCs of chickens administered PBS or LPS. [file 13567_2020_795_MOESM6_ESM.docx]

**
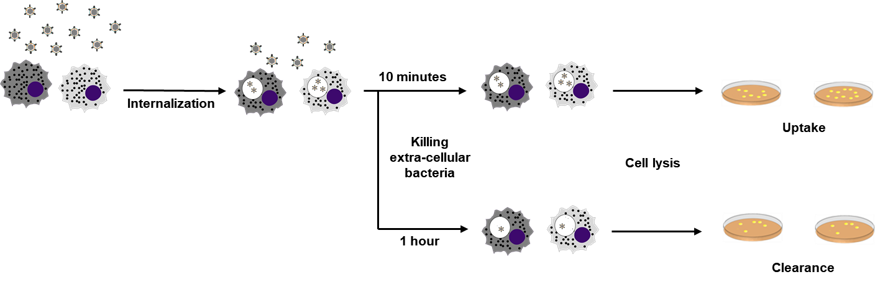
**

Supplement: Supplementary file 7 — Additional file 7. Schematic diagram of the gentamicin protection assay. MRC1loMHCIIhi (filled) and MRC1hiMHCIIlo (open) cells were sorted by a FACS Aria II and coincubated with E. coli K99 for 1 h. After co-incubation, gentamicin (200 μg/mL) was used to kill the extracellular bacteria for 10 min (uptake assay) or 60 min (clearance assay). Cell lysates were incubated overnight on TSA agar to measure the CFU value. [file 13567_2020_795_MOESM7_ESM.docx]
